# Identification of a novel ERF gene, *TaERF8*, associated with plant height and yield in wheat

**DOI:** 10.1186/s12870-020-02473-6

**Published:** 2020-06-08

**Authors:** Lei Zhang, Pan Liu, Jing Wu, Linyi Qiao, Guangyao Zhao, Jizeng Jia, Lifeng Gao, Jianming Wang

**Affiliations:** 1 College of Agronomy, Shanxi Agricultural University, Taigu, China; 2 Institute of Crop Sciences, Chinese Academy of Agricultural Sciences, Beijing, China

**Keywords:** ERF transcription factor, *TaERF8*, Haplotypes, Wheat

## Abstract

**Background:**

Ethylene Responsive Factor (ERF) is involved in various processes of plant development and stress responses. In wheat, several ERFs have been identified and their roles in mediating biotic or abiotic stresses have been elucidated. However, their effects on wheat plant architecture and yield-related traits remain poorly studied.

**Results:**

In this study, TaERF8, a new member of the ERF family, was isolated in wheat (*Triticum aestivum* L.). Three homoeologous *TaERF8* genes, *TaERF8-2A*, *TaERF8-2B* and *TaERF8-2D* (named according to sub-genomic origin), were cloned from the common wheat cultivar Chinese Spring. The three homoeologs showed highly similar protein sequences, with identical AP2 domain. Whereas homoeologs sequence polymorphism analysis allowed the establishment of ten, two and three haplotypes, respectively. Expression analysis revealed that *TaERF8s* were constitutively expressed through entire wheat developmental stages. Analysis of related agronomic traits of *TaERF8-2B* overexpressing transgenic lines showed that *TaERF8-2B* plays a role in regulating plant architecture and yield-related traits. Association analysis between *TaERF8-2B* haplotypes (*Hap-2B-1* and *Hap-2B-2*) and agronomic traits showed that *TaERF8-2B* was associated with plant height, heading date and 1000 kernel weight (TKW). The *TaERF8-2B* haplotypes distribution analysis revealed that *Hap-2B-2* frequency increased in domesticated emmer wheat and modern varieties, being predominant in five major China wheat producing zones.

**Conclusion:**

These results indicated that *TaERF8s* are differentially involved in the regulation of wheat growth and development. Haplotype *Hap-2B-2* was favored during domestication and in Chinese wheat breeding. Unveiling that the here described molecular marker *TaERF8-2B*-InDel could be used for marker-assisted selection, plant architecture and TKW improvement in wheat breeding.

## Background

The APETALA2/Ethylene Responsive Factor (AP2/ERF) superfamily, one of the largest transcription factor families in the plant kingdom [1], is widely involved in various regulatory events, including plant development, plant defense as well as response to environmental stimuli [2]. The AP2/ERF superfamily is characterized by the AP2 domain, which was first identified in regulator proteins for *Arabidopsis* flower development. Based on the number of AP2 domains and other structural features, the AP2/ERF superfamily is divided into four families: ERF, AP2, RAV, and the soloist [3, 4]. Among them, the ERF family can be further divided into two subfamilies: ERF and DREB [2]. The ERF subfamily is characterized by two characteristic conserved amino acid residues in the β-sheet of the AP2 domain, namely the 14th Ala (A14) and the 19th Asp (D19), which distinguish the ERF subfamily from the DREB subfamily [5].

Transcription factors of the ERF subfamily participate in a variety of regulatory events. To date, a large number of ERF genes have been shown to enhance tolerance to abiotic stress or resistance to multiple diseases when over-expressed in transgenic plants, making them ideal candidates for crop improvement [6]. The ectopic expression of the tomato ERF genes *JERF1* and *JERF3* in rice improves tolerance to drought [7, 8]. Overexpression of tomato *OPBP1* confers resistance to fungal and bacterial pathogens of transgenic tobacco plants [9]. Likewise, tobacco *NtERF5* overexpression enhances resistance to tobacco mosaic virus [10]. In addition, constitutive expression of rice ERF gene *Sub1A* enhances submergence tolerance [11], while other rice ERF genes such as *SNORKEL1* and *SNORKEL2* improve the adaptability of transgenic plants to deep water [12]. So far, several wheat ERF genes have been reported to be involved in stress responses. Over-expression of *TaERF1* and *TaERF3* enhance drought tolerance, while *TaPIE1* improves freezing stress tolerance of transgenic wheat plants [6, 13, 14]. Furthermore, wheat plants overexpressing *TaPIE1* also show increased resistance to necrotrophic pathogen *Rhizoctonia cerealis* [14]. Apart from responding to abiotic or biotic stresses, their roles in regulating growth and developmental processes have also been elaborated in previous studies [2, 6]. *ERF BUD ENHANCER* (*EBE*), an ERF gene in *Arabidopsis*, has been reported to affect shoot branching and axillary bud outgrowth [15]. In rice, the *MULTI*-*FLORET SPIKELET1* (*MFS1*) gene is reported to regulate spikelet meristem determinacy and floral organ identity [16]. *OsEATB*, another rice ERF gene, restricts internode elongation, resulting in reduced plant height and panicle length [17]. Overexpression of *AP37* increases grain filling rate as well as grain yield in rice [18]. In barley, *com2* participates in spike branching, which alters inflorescence architecture, resulting in more grain per spike and higher yield [19]. To date, only a few ERFs have been characterized from wheat, and researches have mainly focused on their roles in defending against pathogen attack or abiotic stress responses [20–22]. In addition, only two studies have shown that ERF genes such as *WFZP* of hexaploid wheat and *branched head*^*t*^ (*bh*^*t*^) of tetraploid wheat play important roles in regulating plant development [23, 24]. Therefore, it was advisable to identify and characterize novel ERF genes involved in wheat development, for genetic enrichment and enhancement of genetic-driven wheat breeding.

Here, we isolated and characterized a novel ERF member, TaERF8, in wheat (*Triticum aestivum* L.). Our findings suggest that TaERF8 is involved in wheat growth and development, including regulation agronomic traits such as plant height, heading date, kernel width and TKW. In this study, the genomic sequences of *TaERF8*s in different wheat accessions were analyzed, being identified several haplotypes for each of the homoeologous genes. Molecular marker *TaERF8-2B*-InDel was developed for marker-assisted selection in wheat breeding and for wheat population studies. Haplotype *Hap-2B-2* was unveiled as selected during Chinese wheat breeding. Association analysis showed that *TaERF8-2B* plays a role in plant architecture and yield-related traits.

## Results

### Cloning and characterization of *TaERF8s*

**Fig. 1:**
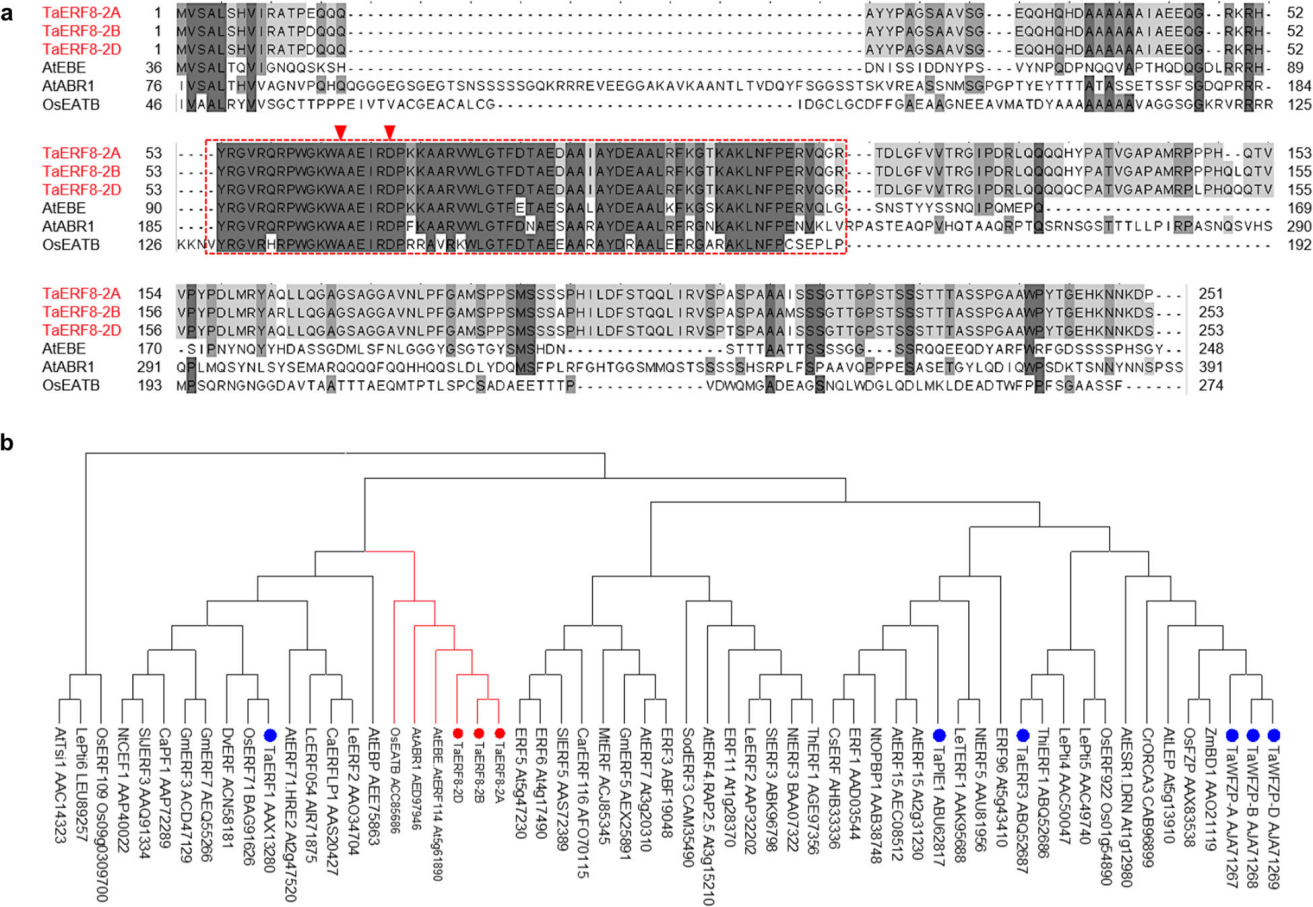
Wheat TaERF8s belong to the ERF family. **a** Alignment of ERFs from different plant species; The conserved AP2 domains were marked in red rectangle; typical amino acid residues at the14th (**a**) and the 19th (**d**) positions of β-sheet were indicated by red triangle. **b** Phylogenetic tree of ERF proteins. TaERF8-2A, TaERF8-2B and TaERF8-2D were marked with red dots; blue dots indicated some of the ERFs identified in wheat

TaERF8 was screened from the sequence library of a project on the introduction of large-scale wheat transcription factor genes into rice [25]. General primer P-G was used to obtain both the coding and genomic sequences of *TaERF8*, allowing the isolation of three *TaERF8* homoeologous genes from the common wheat cultivar Chinese Spring. Isolated homoeologs were designated as *TaERF8-2A*, *TaERF8-2B* and *TaERF8-2D* according to their genomic origins (Additional file 1: Figure S1)*.* The *TaERF8* homoeologs encoded highly homologous proteins (> 96% identity) with lengths of 251, 253 and 253 amino acids, respectively. All three homoeologs shared the same sequence within the AP2 domain, implying that they may have similar functions in wheat, including the ERF subfamily characteristics A14 and D19 in their AP2 domain (Fig. 1a). These similarities allowed their classification as ERF subfamily members, being further grouped into the B4 family according to Sakuma [26]. TaERF8s and ERFs orthologs from *Arabidopsis thaliana* and *Oryza sativa* were grouped into the same clade in the inferred neighbor-joining phylogenetic tree (Fig. 1b), suggesting that TaERF8s might possess similar functions compared to these ERFs.

### Sequence polymorphism and molecular markers development

**Fig. 2:**
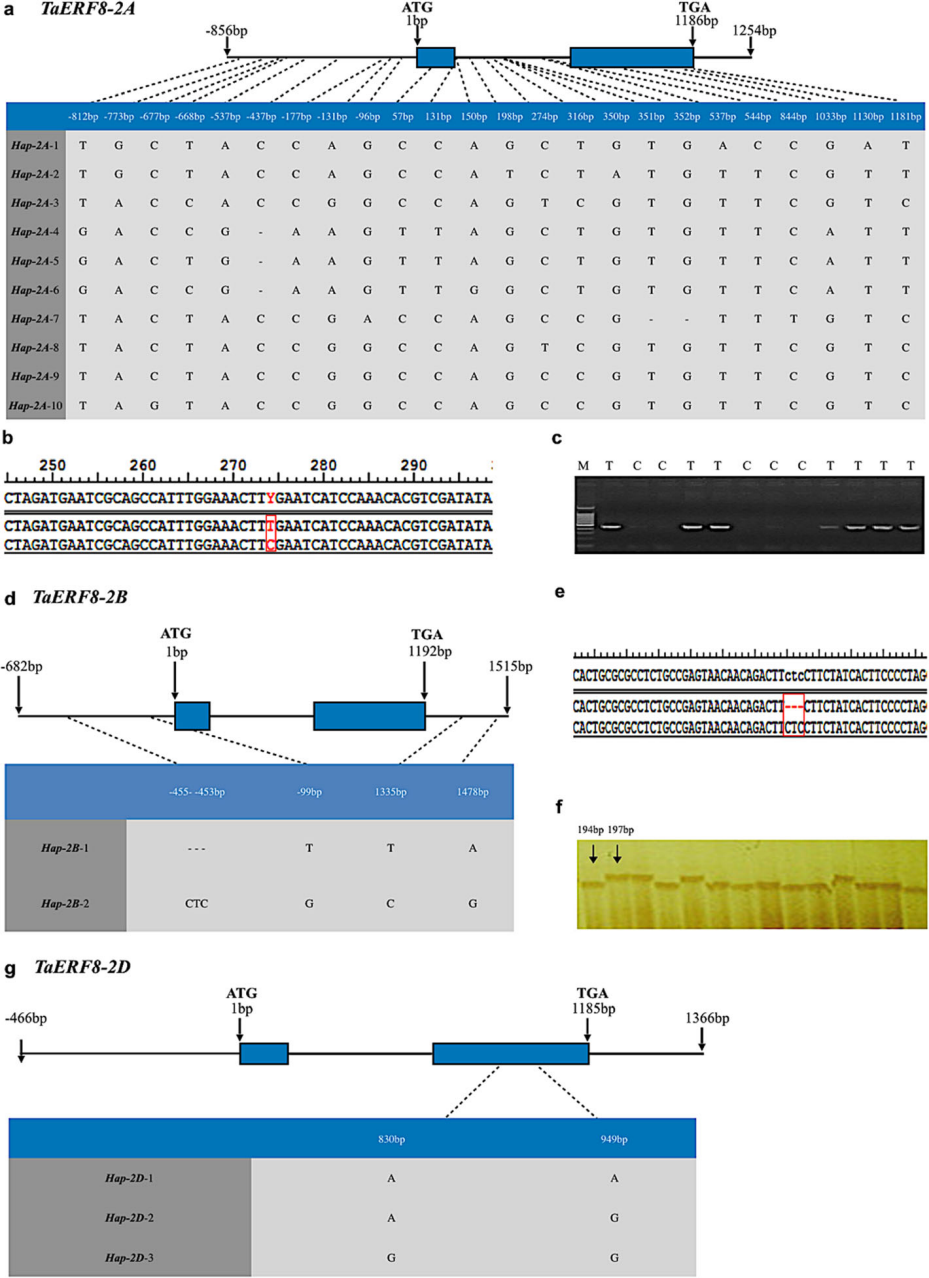
Sequence polymorphism and molecular marker of *TaERF8s.***a** SNPs found in *TaERF8-2A* among different wheat accessions. **b** Molecular marker *TaERF8-2A*-SNP was developed based on the SNP detected at 274 bp (T/C). **c** PCR products were obtained by screening the YZ1/NX188 population using marker *TaERF8-2A*-SNP. M: Marker III. **d** SNPs and InDel found in *TaERF8-2B* among different wheat accessions. **e** Molecular marker *TaERF8-2B*-InDel was developed based on the polymorphic InDel (−−−/CTC) site. **f** Products were obtained by screening the H10/L14 population [27] using marker *TaERF8-2B*-InDel. **g** SNPs found in *TaERF8-2D* among different wheat accessions

Gene-specific primers P-gA, P-gB and P-gD (see Methods section) were employed to amplify the genomic and flanking sequences of *TaERF8-2A*, *TaERF8-2B* and *TaERF8-2D*, respectively, in different wheat accessions (Additional file 2: Table S1) to detect sequence polymorphisms in the three genomes. For *TaERF8-2A*, a total of 24 single nucleotide polymorphism (SNPs) sites were detected, forming 10 haplotypes, *Hap-2A-1* ~ *10* (Fig. 2a)*.* Of the 24 SNPs, six were found within exons, nine in the intron, and the rest were found at the promoter region. Molecular marker *TaERF8-2A*-SNP was developed on the SNP detected at 274 bp (T/C) for mapping *TaERF8-2A* to Yanzhan1 (YZ1)/Neixiang188 (NX188) [28] genetic map (Fig. 2b and c). For *TaERF8-2B*, no SNPs were detected in the coding region, whereas three SNPs (at -99 bp, 1335 bp, and 1478 bp) and a 3-bp InDel (from -455 bp to -453 bp) were identified in upstream and downstream regions*.* With those four polymorphisms, two haplotypes were established, namely *Hap-2B-1* and *Hap-2B-2* (Fig. 2d)*.* Molecular marker *TaERF8-2B*-InDel was developed on the 3-bp InDel site to distinguish the two haplotypes (Fig. 2e and f). And finally, in *TaERF8-2D*, two SNPs at 830 bp and 949 bp were found, identifying three haplotypes, *Hap-2D-1*, *Hap-2D-2* and *Hap-2D-3* (Fig. 2g) (Additional file 3: Figure S2)*.*

### Chromosomal locations of *TaERF8s*

**Fig. 3:**
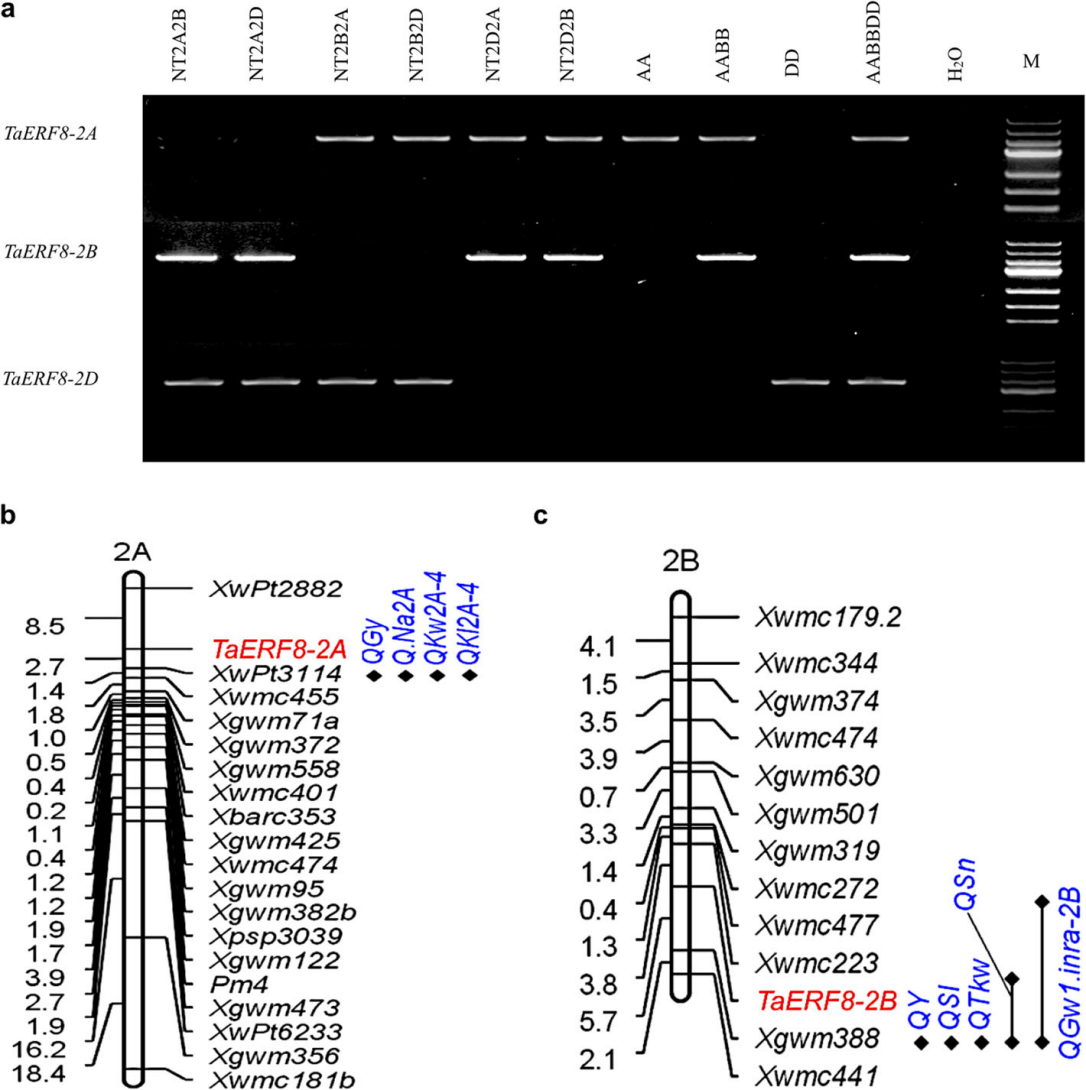
Chromosomal localization of *TaERF8s.***a** Localization of *TaERF8s* on homoeologous group 2 using Chinese Spring nullisomic-tetrasomic lines, diploid, tetraploid and hexaploid wheat. AA: *T.urartu*; AABB: *T.dicoccoides*; DD: *A.tauschii*; AABBDD: Chinese Spring; M: DNA Marker III. **b***TaERF8-2A* was mapped to chromosome 2A flanked by *Xwpt2882* and *Xwpt3114.***c***TaERF8-2B* was mapped to chromosome 2B flanked by *Xwmc223* and *Xgwm388.* Locations of *TaERF8-2A* and *TaERF8-2B* were marked in red, black diamonds indicate QTL associated with agronomic traits reported previously, *QGy* [27]; *QNa2A* [27, 29]; *QKw2A-4* and *QKl2A-4* [30]; *QY* [31]; *QSl* and *QTkw* [32]; *QSn* [33]; *QGw1.inra-2B* [34]

The chromosomal locations of the three *TaERF8* homoeologous genes were verified using the Chinese Spring nullisomic-tetrasomic lines and wheat accessions including diploid, tetraploid and hexaploid wheat (Additional file 2: Table S1). The PCR results showed that gene-specific primers for *TaERF8-2A*, *TaERF8-2B* and *TaERF8-2D* were amplified only in the presence of chromosomes 2A, 2B and 2D, respectively (Fig. 3a). The *TaERF8-2A* gene was mapped to a genetic region flanked by *Xwpt2882* (8.5 cM) and *Xwpt3114* (2.7 cM) by scanning the recombinant inbred line (RIL) population of 199 lines developed by YZ1/NX188 crossing [28] using marker *TaERF8-2A*-SNP (Fig. 3b). *TaERF8-2B* was mapped to a region flanked by *Xwmc223* (3.8 cM) and *Xgwm388* (5.7 cM) by scanning the doubled haploid (DH) population derived from Hanxuan10 (H10)/Lumai14 (L14) crossing using marker *TaERF8-2B*-InDel (Fig. 3c).

### Expression patterns of *TaERF8s* in wheat

**Fig. 4:**
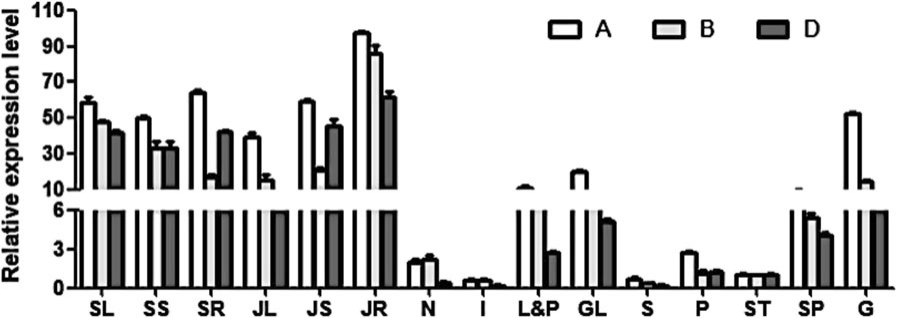
Expression patterns of *TaERF8s* in wheat. SL: Leaf at seedling stage; SS: Stem at seedling stage; SR: Root at seedling stage; JL: Leaf at jointing stage; JS: Stem at jointing stage; JR: Root at jointing stage; N: node; I: internode; L&P: lemma & palea; GL: glume; S: spike; P: pistil; ST: stamen; SP: spikelet; G: grain. A: *TaERF8-2A*, B: *TaERF8-2B* and D: *TaERF8-2D*. The error bars represent SD from three replicates

The expression analysis was performed using common wheat Chinese Spring plants. Gene-specific primers P-qA, P-qB and P-qD were used to detect the expression patterns of *TaERF8s* in various tissues at different developmental stages by quantitative real-time PCR (qRT-PCR). As shown in Fig. 4, transcripts of *TaERF8-2A*, *2B* and *2D* were identified in all the tested tissues, with higher levels of expression in leaf at the seedling stages and in root at the jointing stage. The expression level in tissues like internodes and spike was much lower. The constitutive expression patterns indicated that *TaERF8s* might play active roles through the entire growth cycle of wheat.

### Association of *TaERF8-2B* haplotypes with agronomic traits

**Fig. 5:**
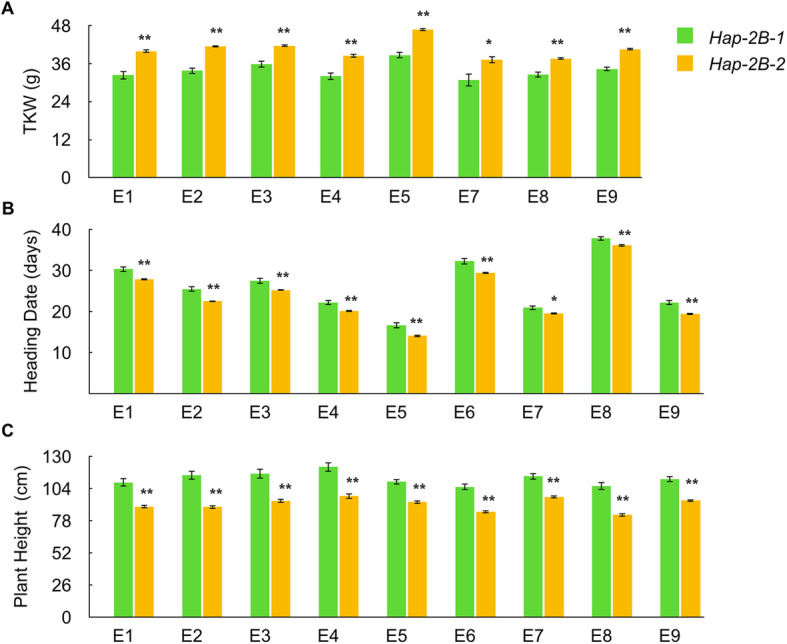
Phenotypic comparisons of two *TaERF8-2B* haplotypes in nine environments. Traits were TKW (**a**), heading date (**b**) and plant height (**c**). E1 to E9 indicated the environments of 2012-BJ, 2012-XX, 2012-JZ, 2012-LY, 2014-XX, 2014-BJ, 2015-XX, 2015-BJ and 2015-JZ, respectively. Heading Date (days from April 1st). The error bars represent SE; * *P* < 0.05, ** *P* < 0.01

The association analysis between *TaERF8-2B* haplotypes and agronomic traits was based on accessions summarized in sample set 1 (Additional file 4: Table S2). Phenotypic data was collected from Beijing (BJ), Luoyang (LY), Xinxiang (XX) and Jiaozuo (JZ), respectively, during the growing seasons of 2012, 2014 and 2015. Significant associations were found between *TaERF8-2B* haplotypes and agronomic traits such as plant height, heading date and TKW (Additional file 5: Table S3). In addition, significant differences were observed for the two haplotypes of *TaERF8-2B* (Additional file 6: Table S4). Accessions possessed *Hap-2B-2* exhibited shorter plant height (16.5 cm-25.7 cm), earlier heading date (1.4d-3.0d) and higher TKW (5.1 g-8.0 g) than those possessed *Hap-2B-1* (Fig. 5)*.* These results implied that *TaERF8-2B* might be involved in multiple processes of wheat growth and development.

### *TaERF8-2B* underwent selection in Chinese wheat breeding

**Fig. 6:**
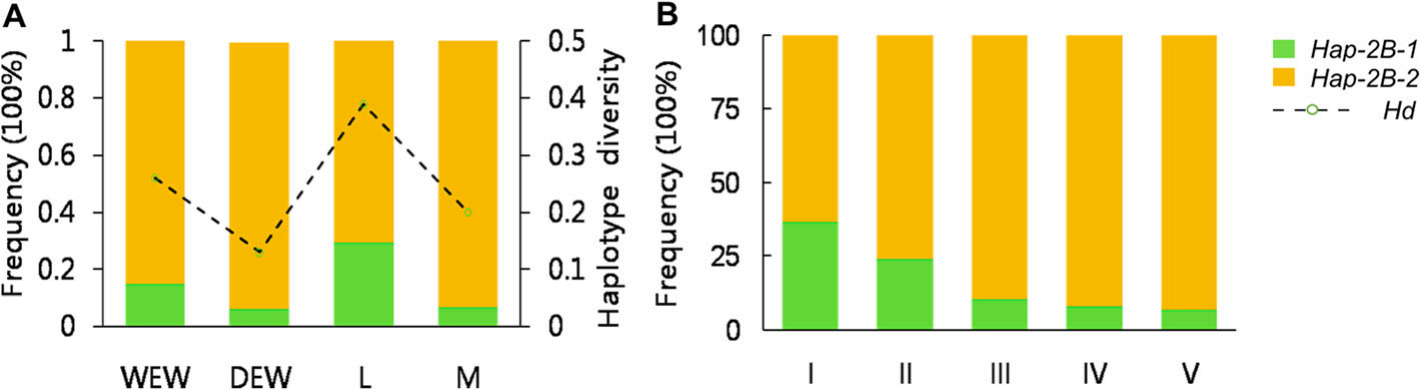
Favored haplotype was selected during domestication and Chinese wheat breeding. **a** Frequency of *TaERF8-2B* haplotypes in sample set 1 and 2. WEW: wild emmer wheat; DEW: domesticated emmer wheat; L: landraces; M: modern varieties; Hd: haplotype diversity. **b** Geographic distribution of *TaERF8-2B* haplotypes in five major wheat-producing zones in China. Zone I: the Northeastern Spring Wheat Zone; II: the Northern Winter Wheat Zone; III: the Huanghuai River Winter Wheat Zone; IV: the middle and lower reaches of Yangtze River Winter Wheat Zone; IV: the Southwestern Winter Wheat Zone

To determine whether *TaERF8-2B* had undergone selection pressure, we investigated the frequency and distribution of the two haplotypes based on sample set 1 and 2 (Additional file 7: Table S5). The results showed that the haplotype diversity of *TaERF8-2B* decreased in domesticated emmer wheat and modern varieties, suggesting that *TaERF8-2B* was selected during the domestication and Chinese wheat breeding. The proportion of *Hap-2B-1* decreased from 15% of wild emmer wheat to 6% of domesticated emmer wheat, and the proportion decreased significantly from 29.2% of wheat landraces to 6.8% of modern varieties (Fig. 6a). On the contrary, the proportion of *Hap-2B-2* increased in both domesticated emmer wheat and modern varieties, indicating that *Hap-2B-2* experienced a positive selection and was the favored haplotype. We further investigated the geographic distribution of *TaERF8-2B* haplotypes based on sample set 3 (Additional file 8: Table S6) from five major wheat-producing zones in China, including the Northeastern Spring Wheat Zone (I), the Northern Winter Wheat Zone (II), the Huanghuai River Winter Wheat Zone (III), the middle and lower reaches of Yangtze River Winter Wheat Zone (IV) and the Southwestern Winter Wheat Zone (V) (Fig. 6b). The results showed that in all five zones, the frequency of *Hap-2B*-2 was higher than that of *Hap-2B*-1, indicating that *Hap-2B*-2 was selected and the dominant haplotype in the five Zones. The frequency of *Hap-2B*-2 was the highest in Zone V (93%), followed by Zones IV, III, and II, and was the lowest in Zone I (63.6%) where the latitude is high. In addition, the frequency of *Hap-2B-2* in five wheat-producing zones increased in a stepwise manner from north to south, while the frequency of *Hap-2B-1* displayed the opposite trend.

### Overexpression of *TaERF8-2B* in wheat influences overall plant development

**Fig. 7:**
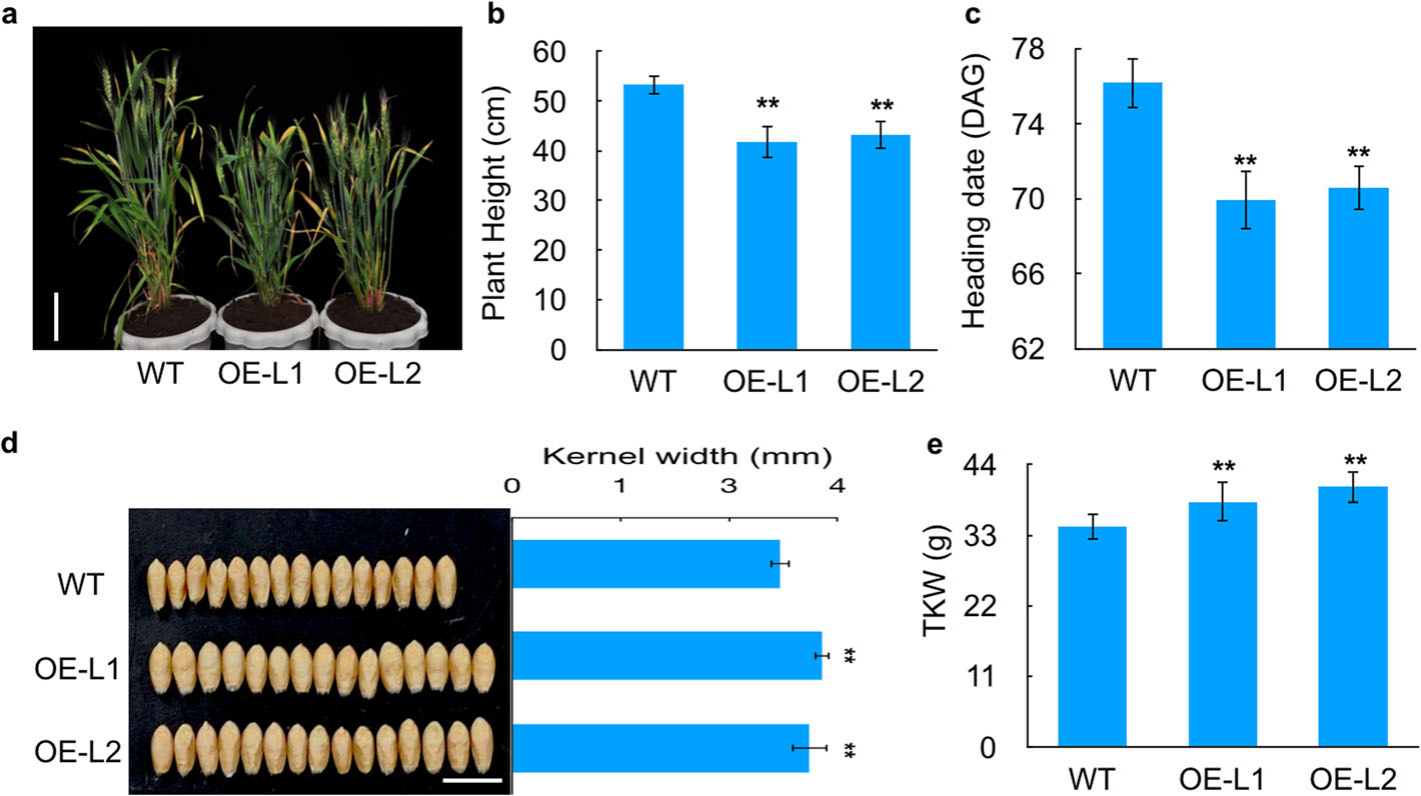
Phenotypic analysis of *TaERF8-2B-*overexpression (OE) transgenic wheat. **a** Plant morphology of WT and two independent OE transgenic lines, scale bar:10 cm; comparison of OE transgenic lines and WT in plant height **b**; heading date (**c**); kernel width, scale bar:10 mm (**d**); TKW (**e**). WT: wild type; data were means ± SD of 20 plants; ^**^P < 0.01

To characterize the functions of *TaERF8-2B*, the coding sequence of *TaERF8-2B* was transformed into the common wheat cultivar Kenong199. Four independent *TaERF8-2B-*overexpression transgenic lines were obtained, and two transgenic lines with different expression levels were used for phenotypic identification (Additional file 9: Figure S3). Several agronomic traits including plant height, heading date, kernel width and TKW were investigated between the *TaERF8-2B* transgenic lines and wild type. The results showed that the *TaERF8-2B* transgenic lines exhibited significantly reduced plant height, earlier heading date, increased TKW and wider kernel width compared to the wild type (Fig. 7). The *TaERF8-2B* transgenic lines exhibited significant changes in the architecture and development of wheat, indicating *TaERF8-2B* might have multiple effects in modulating wheat growth.

## Discussion

### TaERF8 is a novel member of the wheat ERF family

Members of ERF family, which belongs to the AP2/ERF superfamily, are involved in regulation of various processes of plant development and stress responses [5]. To date, a variety of wheat ERFs have been identified and characterized in previous studies [20–24], focusing primarily on their roles in biotic and abiotic stress responses, but little attention has been paid to their roles in relation to yield-related traits. Here, we reported three TaERF8s, as members of wheat ERF by sequence homology and protein similarity, and focused on functions related to plant architecture and growth. In this study, *TaERF8-2A* was mapped to a pericentromeric region of chromosome 2A (flanked by *Xwpt2882* (8.5 cM) and *Xwpt3114* (2.7 cM)), where quantitative trait loci (QTL) for several important agronomic traits were found in previous studies [27, 29, 30], including *QGy* for grain yield [27], *QKw2A-4 for* kernel width and *QKl2A-4 for* kernel length [30]. Besides, marker *Xwpt3114* has been reported to be associated with flowering time and Na + exclusion [27, 29]. *TaERF8-2B* was also mapped to a pericentromeric region of chromosome 2B (flanked by *Xwmc223* (3.8 cM) and *Xgwm388* (5.7 cM)). In this region of chromosome 2B, several QTLs controlling yield-related traits, such as spike length [32], TKW [32, 34], number of fertile spikelets [33] and high yield [31] were detected. As indicated in the results sections, we found that *TaERF8-2B* was related to plant height, heading date and TKW, being the first time that a link between wheat ERF genes and agronomic traits has been established in this chromosomal interval.

Gene expression studies showed that *TaERF8*s were constitutively expressed in multiple tissues at different developmental stages, suggesting their roles as pleiotropic genes in multiple developmental processes. It has been reported that overexpression of the rice ERF gene *OsEATB* results in reduced plant height, and panicle length while increased number of tillers and spikelets [17]. Another *Arabidopsis* ERF gene, *EBE*, has impact on shoot branching, and the overexpression plants stimulate axillary bud formation and outgrowth [15]. The three TaERF8s here reported were clustered into the same clade as *OsEATB* and *EBE*. The overexpression of *TaERF8-2B* in wheat caused a range of changes, including reduced plant height, earlier heading date, wider kernel width and increased TKW per plant. Together, these data show that *TaERF8* is a novel, pleiotropic gene in the wheat ERF gene family, involved in the regulation of wheat growth and development.

### *TaERF8-2B* is a regulatory factor in wheat growth and development

Wheat is one of the most important food crops in the world, and the constant pursuit of increased yield potential has become a major goal of breeding in China in recent decades [35]. As one of the three determinants of grain yield, TKW is believed to have a great impact on yield and is largely determined by kernel size, and in traits associated with kernel size, the kernel width displays the highest association with kernel weight [36–38]. In this study, association analysis and transgenic plants with higher TKW both confirmed that *TaERF8-2B* affects TKW, and the increased TKW in transgenic plants might be due to wider kernel width compared to the wild type.

Plant height is not only a decisive factor in plant architecture but also an important agronomic trait directly linked to yield potential [39]. Genes involved in GA signaling and biosynthesis have been used in breeding to modify and produce crops with reduced height, increased yield production and other beneficial traits [40–45], which can be best exemplified in the Green Revolution [46]. Previous studies have shown that some ERFs are related to GA in regulating plant height [11, 12, 17]. The rice ERF gene *OsEATB* restricts the internode elongation by down-regulating a gibberellin biosynthetic gene, which results in reduced plant height and panicle length, and increased tiller and spikelet number in *OsEATB-*overexpressed plants [17]. *Sub1A*, another rice ERF gene, restricts the response to GA through elevating the expression of *SLR1* and *SLRL1* [11], while *SK1* and *SK2*, also encode ERFs, trigger internode elongation via GA in response to flooding [12]. Here by sequence analysis, we identified a gibberellin-responsive element (GARE-motif) in the promoter region of *TaERF8-2B*. However, whether *TaERF8-2B* is involved in GA-related plant height regulation still needs further investigation. Taken together, overexpressing *TaERF8-2B* led to a reduced height and changes in yield-related agronomic traits, suggesting its role in regulating plant architecture and growth.

### *TaERF8-2B* has undergone selection during wheat breeding

Domesticated crops, including wheat, have undergone strong human selection aimed at developing cultivars with favorable traits to adapt to a wider range of environmental conditions [47]. Hexaploid bread wheat has experienced two major selection events, one is the selection from wild emmer wheat to domesticated emmer wheat during the domestication process, and the other is the selection that involves crop improvement from landraces to modern varieties during wheat breeding process [48]. In this study, we identified that *TaERF8-2B* had undergone the two selective events. The proportion of *Hap-2B-1* decreased from 15% of wild emmer wheat to 6% of domesticated emmer wheat, and the proportion decreased significantly from 29.2% of wheat landraces to 6.8% of modern varieties. On the contrary, the proportion of *Hap-2B-2* increased in both domesticated emmer wheat and modern varieties. The geographic distribution of *TaERF8-2B* haplotypes showed that the frequency of *Hap-2B-2* was higher than that of *Hap-2B-1* in five major wheat-producing zones of China, indicating that *Hap-2B*-2 was selected. Association analysis of *TaERF8-2B* haplotypes with agronomic traits showed that *Hap-2B-2* was significantly associated with reduced plant height and higher TKW, which were favorable traits for wheat breeding. All of these results together indicated that *Hap-2B-2* had undergone positive selection, being the favored haplotype during Chinese wheat breeding. In addition, we noted a regional adaptation selection of *Hap-2B-2*. From northern to southern China, the frequency of *Hap-2B-2* increased by more than 10%, while the frequency of *Hap-2B-1* displayed the opposite trend. In general, varieties planted in the North regions of China possess enhanced photoperiod sensitivity, while varieties grown in southern China with lower latitude often exhibit reduced photoperiod sensitivity [49]. In this study, earlier heading date was observed for wheat overexpressing *TaERF8-2B* under long-day conditions, indicating that *TaERF8-2B* may affect the heading date. However, whether *TaERF8-2B* is involved in relevant pathways that modulate photoperiod sensitivity requires a more comprehensive analysis.

Given the large and complex genomes of wheat, knowledge about the function of specific gene associated with agronomic traits is important for the efficient selection of new haplotypes in breeding [50]. Functional markers, an ideal tool for marker-assisted breeding, have been developed for abiotic stress tolerance and agronomic traits including plant height and grain weight [51–53]. In this study, functional analysis of *TaERF8-2B* revealed its role in affecting plant height and yield-related traits, and the functional marker *TaERF8-2B*-InDel was successfully developed, being able to identify a selected haplotype (*Hap-2B-2*). Therefore, utilization of this functional marker could contribute to further improvement of plant architecture and TKW in wheat.

## Conclusions

Three homoeologous genes of *TaERF8*, *TaERF8-2A*, *TaERF8-2B* and *TaERF8-2D* were cloned from the common wheat cultivar Chinese Spring. Sequence analysis showed that the three homoeologs were highly similar, possessing the same AP2 domain, and belonging to the ERF subfamily. Expression analysis revealed that *TaERF8s* were constitutively expressed through entire wheat developmental stages. Polymorphic sites were detected in *TaERF8-2B*, which allowed the development of breeding-assistant markers and the identification of haplotype selection in Chinese wheat breeding (*Hap-2B-2*). Association analysis revealed that *TaERF8-2B* was significantly associated with plant height, heading date and TKW. In summary, the newly developed molecular marker *TaERF8-2B*-InDel may be useful for marker-assisted selection, plant architecture and TKW improvement in wheat breeding.

## Methods

### Plant materials and growth conditions

Common wheat cultivar Chinese Spring was used to clone the *TaERF8-2A*, *TaERF8-2B* and *TaERF8-2D* genes*.* A total of 42 different wheat accessions including diploid, tetraploid and hexaploid wheat were used for polymorphism analysis of *TaERF8s* (Additional file 2: Table S1). The 42 accessions were planted in the experimental field of the Institute of Crop Science, the Chinese Academy of Agricultural Sciences, Beijing (39°N, 116°E) in a 2-row 2 m plot with 50 cm between rows.

Association analysis of *TaERF8-2B* haplotypes with agronomic traits was performed using sample set 1, which contains 367 wheat accessions, including 89 landraces and 278 modern varieties (Additional file 4: Table S2). The sample set 1 was planted in the growing seasons of 2012, 2014 and 2015 at four locations, viz., BJ (40°N, 116°E), XX (35°N, 113°E), LY (33°N, 111°E) and JZ (35°N, 113°E) in a 4-row 2 m plot with 25 cm between rows. Field managements, including irrigation, fertilization and pest control were carried out according to local production conditions. Phenotypic assessment of plant height, heading date and TKW was performed under 9 environments (E1 to E9) (Additional file 6: Table S4). E1 to E9 indicated the environments at BJ, XX, JZ and LY in 2012, XX, BJ in 2014 and XX, BJ and JZ in 2015, respectively. For each accession, plant height was averaged by measuring 8 individual plants, heading date was recorded when 50% plants showed fully emerged spikes, and TKW was calculated by 5 times of the weight of 200 grains.

Frequency of *TaERF8-2B* haplotypes was calculated based on sample set 1 and 2. The sample set 2 consisted of 107 wild and 32 domesticated tetraploid wheat (Additional file 7: Table S5). Sample set 3 (Additional file 8: Table S6) was used to illustrate the geographic distribution of *TaERF8-2B* haplotypes in different wheat production zones in China. These accessions were planted in 2015 growing season at XX (35°N, 113°E), and field managements were conducted according to local production conditions. Accessions of sample set 2 were planted as single 50 cm × 2 m plots and sample set 3 were in a 2-row 25 cm × 2 m plots.

The RIL population of 199 lines developed by YZ1/NX188 crossing [28], and DH population derived from H10/L14 crossing [54] were used for linkage mapping. The RIL population was grown at Luoyang (33°N, 111°E) in 2015 growing season. Thirty seeds of each line were sown in a 2-row plot of 2 m in length. The p*Ubi: TaERF8-2B* transgenic lines used in this study were in the wheat cultivar Kenong199 background. Kenong199 and transgenic plants were cultured in plastic pots filled with Pindstrup substrate (Pindstrup, Denmark), a peat-based potting mix, and placed in a controlled growth chamber under the 16 h light / 8 h dark photoperiod at 22 °C. Watering was repeated every two or three days. The DH population derived from H10/L14 crossing was kindly provided by Dr. Ruilian Jing of the Institute of Crop Science, Chinese Academy of Agricultural Sciences, and the other plant materials used in this study were provided by the Key Laboratory of Crop Gene Resources and Germplasm Enhancement of the Institute of Crop Science, Chinese Academy of Agricultural Sciences.

### Data analysis

In association analysis, 367 accessions were classified into group 1 and group 2 according to the two haplotypes of *TaERF8-2B*, respectively. Genotypic and phenotypic data were imported and analyzed using SPSS 19.0 software (SPSS Inc., USA). Correlation between *TaERF8-2B* haplotypes and agronomic traits was analyzed, the mean comparison of agronomic traits between the two haplotypes was performed, and the statistical t-test was applied to confirm the significance. Association was considered significant at *P* < 0.05. Frequency of *TaERF8-2B* haplotypes was calculated based on sample set 1 and 2. Haplotype diversity of *TaERF8-2B* was calculated for sample set 2 using Powermarker software [55].

### Cloning and chromosomal locations of the three *TaERF8* genes

In this study, the general primer pair P-G (5′-CCGTATCACCACCTCATC-3′ and 5′-TGCGTATTCCTCATCTACTG-3′) was used to amplify the genomic and full-length cDNA sequences of *TaERF8* from Chinese Spring. PCR amplification was performed in 25 μL volume including 1 μL 100 ng/μL cDNA or genomic DNA, 7.5 pmol of each primer, 5 μL 2 mM dNTPs, 12.5 μL 2 × PCR buffer for KOD FX Neo, and 0.5 μL (1.0 U/μL) KOD FX Neo DNA Polymerase (Toyobo, Japan). The amplification procedure was as follows: initial denaturation at 94 °C for 2 min, followed by 33 cycles of denaturation at 98 °C for 10 s, annealing at 58 °C for 30 s, and extension at 68 °C for 30 s–1 min, with a final extension at 68 °C for 10 min. The amplified PCR product was cloned into pEASY-Blunt Zero cloning vectors (Transgen, China). Based on the sequence differences among the A, B and D wheat genomes of *TaERF8*, three gene-specific primers, P-gA (5′-CCAAATGTTGAGTGACTTG-3′ and 5′-TGCGTATTCCTCATCTACTG-3′), P-gB (5′-GAATGAACGGGAAATGTTATCCAT-3′ and 5′-GAAACGATAAACGATTAGACCA-3′) and P-gD (5′-CAGACTTCTCCTTCTATCACAT-3′ and 5′-AAACAAAACAAGAGATTTAGATGA-3′), were designed. The amplification procedure was as follows: denaturation at 94 °C for 2 min, followed by 33 cycles of denaturation at 98 °C for 10 s, annealing at 60 °C for 30 s, and extension at 68 °C for 80 s, with a final extension at 68 °C for 10 min. The gene-specific primers, Chinese Spring nullisomic-tetrasomic lines and wheat species of different ploidy (Additional file 2: Table S1) were then used to determine the chromosomal locations of the three homoeologous genes.

### Gene expression analysis of *TaERF8*

Gene expression analysis of *TaERF8* was performed using common wheat Chinese Spring plants. The Chinese Spring plants were splitted in leaf, stem, root, pistil, spikelet, node, internode, stamen, young spike, glume, lemma and palea at their precise developmental stage from at least six independent plants, mixed and fast frozen at − 80 °C till RNA extraction. Total RNA extraction was performed with Direct-zol™ RNA miniprep kit (Zymo Research, USA) following manufacturer instructions. One microgram of total RNA of each sample was used for first-strand cDNA synthesis using the 5 × All-In-One MasterMix kit (ABM, Canada). Specific primers for *TaERF8-2A* gene (P-qA: 5′-CCAGACCGTGGTGCCGTACC-3′ and 5′-GCCGCCGCGGGAGACGCT-3′), *TaERF8-2B* gene (P-qB: 5′-GACCTCATGCGGTATGCACG-3′ and 5′-TTGTGCCTGAGCTCGACATTG-3′) and the *TaERF8-2D* gene (P-qD: 5′-CGACAGATTGCAGCAACAACAACAGTG-3′ and 5′-CCCGTTGTGCCTGAGCTCGATATA-3′) were used on this first-strand cDNA (2 μl of ten times diluted first-strand cDNA) to determine relative transcript level for each wheat tissue by qRT-PCR. The qRT-PCR was performed using SYBR® Premix Ex Taq (Takara, China). Tubulin gene (Gen Bank accession no TAU76544) was amplified similarly with primer pair P-qT (5′-TCGATGATCTCCAACTCCACCAGT-3′ and 5′-TCGTCGAACTCAGCACCAACTTCT-3′) as the internal reference. The qRT-PCR was performed using TB Green® *Premix Ex Taq*™ (Takara, China). It was performed in a total volume of 10 μL containing 5 μL of TB Green *Premix Ex Taq*, 2 μL of cDNA and 2 pmol of each gene-specific primer. The reaction program was as follows: denaturation at 95 °C for 3 min, followed by 45 cycles at 95 °C for 10 s and 60 °C for 30 s. All qRT-PCR assays were performed in three independent replications and the relative transcript level of each *TaERF8* gene was determined using the 2^-∆∆CT^ method [56].

### Molecular marker development

Nucleotides of coding and flanking regions in *TaERF8-2A*, *TaERF8-2B* and *TaERF8-2D* were screened among different wheat accessions (Additional file 2: Table S1). Sequences alignment was conducted using SeqMan (DNASTAR Lasergene 7.1.0). Molecular markers were then designed accordingly based on polymorphic sites detected in *TaERF8-2A* and *TaERF8-2B* genomic sequences. Molecular marker *TaERF8-2A*-SNP (5′-CGCAGCCATTTGGAAACTAT-3′ and 5′-AAGGTGCCGAGCCATACA-3′) was designed based on a SNP detected in the intron of the *TaERF8-2A*. Molecular marker *TaERF8-2B*-InDel (5′-CCTTGTTAGACTCCAAAATGCT-3′ and 5′-CGAGCTCTTCTGAACGCTT-3′) was designed according to the InDel site found upstream of the *TaERF8-2B* start codon. The PCR amplification procedure was as follows: denaturation at 94 °C for 2 min, followed by 33 cycles of denaturation at 98 °C for 10 s, annealing at 56 °C for 30 s, and extension at 68 °C for 15 s, with a final extension at 68 °C for 10 min. Both pairs of molecular markers were used for genetic mapping of the corresponding gene, and the marker *TaERF8-2B*-InDel was further applied for *TaERF8-2B* haplotypes analysis*.*

### Isolation and bioinformatic analysis of protein sequences

The protein sequences of cloned ERFs in wheat and other species including *Arabidopsis* and rice were downloaded from the National Center for Biotechnology Information database (NCBI, http://www.ncbi.nlm.nih.gov) according to their accession number. Multiple sequence alignments of the ERFs were conducted using Clustal X [57] with default parameters and then viewed by the Jalview2 [58]. Phylogenetic analysis was performed with the protein sequences of TaERF8s and the other annotated ERFs from wheat, *Arabidopsis*, maize, rice and some other plant species by a Neighbor-Joining method with set parameters (poisson model, uniform rates and complete deletion) using MEGA 6.0 [59]. The robustness of the phylogenetic construction was tested by bootstrap method with 1000 replications.

## Supplementary information

**Additional file 1: Figure S1.** Nucleotide sequences of *TaERF8-2A*, *TaERF8-2B* and *TaERF8-2D*.

**Additional file 2: Table S1.** Wheat accessions information for *TaERF8s* polymorphism analysis.

**Additional file 3: Figure S2.** Nucleotide sequences of *TaERF8-2A*, *TaERF8-2B* and *TaERF8-2D* haplotypes.

**Additional file 4: Table S2.** The information of sample set 1 and their genotypes of *TaERF8-2B*.

**Additional file 5: Table S3.** Association analysis of *TaERF8-2B* haplotypes and agronomic traits in nine environments.

**Additional file 6: Table S4.** Summary of agronomical traits of sample set 1 in nine environments.

**Additional file 7: Table S5.** The information of sample set 2 and their genotypes of *TaERF8-2B*.

**Additional file 8: Table S6.** The frequencies of *TaERF8-2B* allelic variation in sample set 3.

**Additional file 9: Figure S3.** The expression levels of *TaERF8-2B* in overexpression transgenic wheat lines and wild-type plants.

## Data Availability

All data generated or analyzed during this study are included in this published article [and its additional files].

## Abbreviations

AP2/ERF: APETALA2/Ethylene Responsive Factor
ERF: Ethylene Responsive Factor
TKW: 1000 kernel weight
QTL: Quantitative trait loci
SNP: Single nucleotide polymorphism
InDel: Insertion and Deletion
RIL: Recombinant inbred line
DH: Doubled haploid
