# Supplementary material for: Identification of a novel ERF gene, TaERF8, associated with plant height and yield in wheat
Source: BMC Plant Biol. 2020 Jun 8;20:263. doi: 10.1186/s12870-020-02473-6 (PMC7282131; doi:10.1186/s12870-020-02473-6)
Supplement: Supplementary file 1 — Additional file 1: Figure S1. Nucleotide sequences of TaERF8-2A, TaERF8-2B and TaERF8-2D. [file 12870_2020_2473_MOESM1_ESM.docx]

***TaERF8-2A* cDNA:**

ttatcagtacgttgtccgcgcctaggcaccaaagtccaaagcaacagccatagctcgatctcgatccccggcgcgacgaaagagaaagaagcggcggcaggtcgacaggtcgatcaactaaggtggatccccggaggcatgggaagaggcccctacccgccgacgaggaggaggaacagccgccaccgccgccgtcagcagccaagcacgagcaggtggaggagcagccgtatcaccccctcatcgcgcgcgctctgcagcagcaaggagctgccagcgccggcggaagctcgggagcagatgtggccgacccttccccgtcaccggaggcgtacgcgcagtactactactcggcgcgcgccgaccacgacgccaccgccatggtctccgctctgtcccacgtcatccgcgccacaccggaacagcaacaagcctactaccccgccggatccgccgctgtctcaggagaacagcagcatcagcacgatgcggcggctgccgcggccatcgctgaggaacaagggaggaagcggcactacagaggggtgaggcagcggccatggggaaagtgggcggcggagatccgggaccccaagaaagcggctcgtgtatggctcggcacctttgacacggctgaggacgccgccatcgcctacgacgaagcggcgctgcgcttcaagggcaccaaggccaagctcaacttccccgagcgcgtccagggacgcaccgacctcggcttcgtcgtcacgcgcggcattcccgacagattgcagcaacaacaacactaccccgccaccgtgggggcgccggcaatgcggccaccgccgcaccagaccgtggtgccgtaccctgacctcatgcggtatgcacagctgttgcagggcgctggcagtgccgggggcgctgtcaacctgccgtttggcgccatgtcgcccccgtcgatgtcctcgtcgtcgccgcacatactcgacttctcgacacagcagctcatccgagtgagcccagcgtctcccgcggcggcaatatcgagctcaggcacaacggggccgtccacctcatcgtccacgaccacggcatcgtcgccaggtgctgcatggccgtacactggggagcacaaaaataataaagacccgtgagagatcagtggatcgaaggtgcttgcgatccatcgggacatgtcttagcagtagatgaggaatacgcatgcattcatcatgcaataatgtggctaatatgacatatag

***TaERF8-2B* cDNA:**

ccctccgcgttcttatcagtacgttgtccgcgcctaggcaccaaagtccaaagcaacagccatagctcgatctcgatccccggcgcgacgaaagaaaaagaagcggcggcaggtcgacaggtcgatcaactaaggtggatccccggaggcatgggaagaggcccctacccgccgacgaggaggaggaacagccgccaccgccgccgtcagcagccaagcacgagcaggtggaggagcagccgtatcaccacctcatcgggcgcgctctgcagcagcaaggagctgccagcgccggcggaagctcgggagcagatgtggccgacccttccccgtcaccggaggcgtacgcgcagtactactactcggcgcgcgccgaccacgacgccaccgccatggtctccgctctgtcccacgtcatccgcgccacaccggaccagcaacaagcctactaccccgccggatccgccgctgtctcaggagaacagcagcatcagcacgatgcggcggctgccgcggccatcgctgaggaacaagggaggaagcggcactacagaggggtgaggcagcggccatggggaaagtgggcggcggagatccgggaccccaagaaagcggctcgtgtgtggctcggcacctttgacacggctgaggacgccgccatcgcctacgacgaagcggcgctgcgcttcaagggcaccaaggccaagctcaacttccccgagcgcgtccagggacgcaccgacctcggcttcgttgtcacgcgcggcatacccgacagattgcagcaacaacaacactaccccgccaccgtgggggcgccggcaatgcggccaccgccgcaccagctgcagaccgtggtgccgtaccctgacctcatgcggtatgcacggctgttgcagggcgctggcagtgccgggggcgctgtcaacctgccgttcggcgccatgtcgcccccgtcgatgtcctcgtcggcgccgcacatactcgacttctcgacacagcagctcatccgagtgagcccggcgtctcccgcggcggcaatgtcgagctcaggcacaacggggccgtccacctcatcgtccacgactacggcatcgtcgccaggtgctgcatggccgtacactggggagcacaaaaataataaagactcgtgagagatcaatggatcgaaggtgcttgcgatccatcgggacatgtcttagcagtagatgaggaatacgcatgcattcatcatgcaataaggtgggtaagtgttggtcaattaattttgctggtgaacatttctttccttcttctcatccccaaatttgtttatctaaagctcttgttttgtttatttttactttaataatgggtgattttttttggcgggtaatgggtgactcttaactagctaggtactagttgtactaaattagtgacaagtaacatagaacagagggagaccttgttccttggtctaatcgtttatcgtttctcttctcttttatgaagtgtacataagtcttagatgaaagtacatccatttagtaccaatacctttggattgtagacacaaaactcaaatcaaataattaaatatcagatgactttaggaacattgttt

***TaERF8-2D* cDNA:**

gctcgatctcgatccccggcgcgacgaaagaaaaagaagcggcggcaggtcgacaggtcgatcaactaaggtggatccccggaggcatgggaagaggcccctacccgccgacgaggaggaggaacagccgccaccgccgccgtcagcacccaagcacgagcaggtggaggagcagccgtatcaccacctcatcgcgcacgctctgcagcagcaaggagctgccagcgccggcggaagcccgggagcagatgtggccgacccttccccgtcaccggaggcgtacgcgcagtactactactcggcgcgcgccgaccacgacgccaccgccatggtctccgctctgtcccacgtcatccgcgccacaccggaccagcaacaagcctactaccccgccggatccgccgctgtctcaggagaacagcagcatcagcacgatgcggcggctgcagcggccatcgctgaggaacaagggaggaagcggcactacagaggggtgaggcagcggccatggggaaagtgggcggcggagatccgggaccccaagaaagcggctcgtgtgtggctcggcacctttgacacggctgaggacgccgccatcgcctacgacgaagcggcgctgcgcttcaagggcaccaaggccaagctcaacttccccgagcgcgtccagggacgcaccgacctcggcttcgtcgtcacgcgcggcatacccgacagattgcagcaacaacaacagtgccccgccaccgtgggggcgccggcaatgcggccactgccgcaccagcagcagaccgtggtgccgtaccctgacctcatgcggtatgcacagctgttgcagggcgctggcagtgccgggggcgccgtcaacctgccgttcggcgccatgtcgcccccgtcgatgtcctcgtcgtcgccgcacatactcgacttctcgacacagcagctcatccgagtgagcccgacgtctcccgcggcggctatatcgagctcaggcacaacggggccgtccacctcatcgtccacgactacggcatcgtcgccaggtgctgcatggccgtacactggggagcacaaaaataataaagactcgtgagagatcagtggatcgaaggtgcttgcgatccatcgggacatgtcttagcagtagatgaggaatacgcatgcattcatcatgcaataagctaatatgacatatagctacgtccttgtcaagtgtaactatatcaaccatgcattcatgtgccatagagttcttgcaggaatctggttctgtgctttacgaagtcgtactgttgaaagaaggtgcggtatccacggaattagattgtgccgtcattttcagaaacatgcggaaaatcacccttcagttt

**Additional file 1: Figure S1.** Nucleotide sequences of *TaERF8-2A*, *TaERF8-2B* and *TaERF8-2D.* The full length CDSs of *TaERF8-2A*, *TaERF8-2B* and *TaERF8-2D* are shown above and marked in red.
