# Supplementary material for: Identification of a novel ERF gene, TaERF8, associated with plant height and yield in wheat
Source: BMC Plant Biol. 2020 Jun 8;20:263. doi: 10.1186/s12870-020-02473-6 (PMC7282131; doi:10.1186/s12870-020-02473-6)
Supplement: Supplementary file 2 — Additional file 2: Table S1. Wheat accessions information for TaERF8s polymorphism analysis. [file 12870_2020_2473_MOESM2_ESM.docx]

**Additional file 2: Table S1.** Wheat accessions information for *TaERF8s* polymorphism analysis

| Number | Accession | Species | Genome | Origin |
| --- | --- | --- | --- | --- |
| 1 | UR202 | *T. urartu* | AA | Syria |
| 2 | UR203 | *T. urartu* | AA | Syria |
| 3 | UR206 | *T. urartu* | AA | Syria |
| 4 | MO101 | *T. monococcum* | AA | Unknown |
| 5 | MO102 | *T. monococcum* | AA | Unknown |
| 6 | BO103 | *T. boeoticum* | AA | Unknown |
| 7 | BO104 | *T. boeoticum* | AA | Pakistan |
| 8 | Y590 | *A. speltoides* | BB | Unknown |
| 9 | Y2005 | *A. speltoides* | BB | Syria |
| 10 | Y2180 | *A. speltoides* | BB | Unknown |
| 11 | Y2262 | *A. tauschii* | DD | Unknown |
| 12 | Y2268 | *A. tauschii* | DD | Unknown |
| 13 | Y2280 | *A. tauschii* | DD | Unknown |
| 14 | Y2282 | *A. tauschii* | DD | Armenia |
| 15 | PI 94618 | *T. dicoccum* | AABB | Belarus |
| 16 | PI 94632 | *T. dicoccum* | AABB | Ethiopia |
| 17 | PI 94649 | *T. dicoccum* | AABB | Czechoslovakia |
| 18 | PI 94660 | *T. dicoccum* | AABB | Russian |
| 19 | PI 94671 | *T. dicoccum* | AABB | Afghanistan |
| 20 | PI 190919 | *T. dicoccoides* | AABB | Spain |
| 21 | PI 266841 | *T. dicoccoides* | AABB | United Kingdom |
| 22 | PI 346783 | *T. dicoccoides* | AABB | Hungary |
| 23 | PI 352322 | *T. dicoccoides* | AABB | Asia Minor |
| 24 | PI 352327 | *T. dicoccoides* | AABB | Switzerland |
| 25 | PI 355457 | *T. dicoccoides* | AABB | Germany |
| 26 | PI 355458 | *T. dicoccoides* | AABB | Germany |
| 27 | PI 355459 | *T. dicoccoides* | AABB | Armenia |
| 28 | PI 428018 | *T. dicoccoides* | AABB | Turkey |
| 29 | Chadianhong | *T. aestivum* L. | AABBDD | HeBei, China |
| 30 | Chinese Spring | *T. aestivum* L. | AABBDD | Jiangsu, China |
| 31 | Fuzhuang 30 | *T. aestivum* L. | AABBDD | Shaanxi, China |
| 32 | Hanxuan 10 | *T. aestivum* L. | AABBDD | Shanxi, China |
| 33 | Keyi 26 | *T. aestivum* L. | AABBDD | Beijing, China |
| 34 | Laizhou953 | *T. aestivum* L. | AABBDD | Shandong, China |
| 35 | Lumai 14 | *T. aestivum* L. | AABBDD | Shandong, China |
| 36 | Marquis | *T. aestivum* L. | AABBDD | Canada |
| 37 | Neixiang188 | *T. aestivum* L. | AABBDD | Henan, China |
| 38 | Qinghaidasui | *T. aestivum* L. | AABBDD | Qinghai, China |
| 39 | Opata | *T. aestivum* L. | AABBDD | CIMMYT |
| 40 | Xianyangdasui | *T. aestivum* L. | AABBDD | Shaanxi, China |
| 41 | Yanzhan 1 | *T. aestivum* L. | AABBDD | Henan, China |
| 42 | Zaosui30 | *T. aestivum* L. | AABBDD | Beijing, China |
