# Supplementary material for: Identification of a novel ERF gene, TaERF8, associated with plant height and yield in wheat
Source: BMC Plant Biol. 2020 Jun 8;20:263. doi: 10.1186/s12870-020-02473-6 (PMC7282131; doi:10.1186/s12870-020-02473-6)
Supplement: Supplementary file 3 — Additional file 3: Figure S2. Nucleotide sequences of TaERF8-2A, TaERF8-2B and TaERF8-2D haplotypes. [file 12870_2020_2473_MOESM3_ESM.docx]

***Hap-2A-1*:**

CCAAATGTTGAGTGACTTGGCGTTGAGATTAGTTCATACAGATCTAAAATATGTCATATAAAAGTGTAATAATTTGAGTATTTGGTGCAAAAAAATTGGTATTATCTCTTTTTCGAATATTTTTTGATTTCTTACACACGTGTCACATAGTAAATTTGGTATTATCTCCATTGAATGAACGCGCAATGTTATTCATACATCATCATATTGTAATAATTTGTCTGTTTAGAGTGTAAAAGATATATTACTTCTTGTCCAGACTCCAAAATGCTGAAATTTTGTACAGTACATATTTTGCACTAAGAAATTTAATGGCAATACCGAAATACTCCGTACAAAGTGCTCAGCCAAAGGTGCTAGAACTGGGACACTGCGCGCCTCTGCCGAGTAACAACAGACTTCTCCTTCTATCACTTCCCCTAGCTATCATTAAAGGCGTTCAGAAGAGCTCGTCTCCTCTCTCTCCCTCCGCGTTCTTATCAGTACGTTGTCCGCGCCTAGGCACCAAAGTCCAAAGCAACAGCCATAGCTCGATCTCGATCCCCGGCGCGACGAAAGAGAAAGAAGCGGCGGCAGGTCGACAGGTCGATCAACTAAGGTGGATCCCCGGAGGCATGGGAAGAGGCCCCTACCCGCCGACGAGGAGGAGGAACAGCCGCCACCGCCGCCGTCAGCAGCCCAGCACGAGCAGGTGGAGGAGCAGCCGTATCACCCCCTCATCGCGCACGCTCTGCAGCAGCAAGGAGCTGCCAGCGCCGGCGGAAGCTCGGGAGCAGATGTGGCCGACCCTTCCCCGTCACCGGAGGCGTACGCGCAGTACTACTACTCGGCGCGCGCCGACCACGACGCCACCGCCATGGTCTCCGCTCTGTCCCACGTCATCCGCGCCACACCGGAACAGCAACAAGCCTACTACCCCGCCGGATCCGCCGCTGTCTCAGGAGAACAGCAGCATCAGCACGATGCGGCGGCTGCCGCGGCCATCGCTGAGGAACAAGGTAATCAACAATAGCTGCTAGCATCCATCGATGTATCCAAACGTAGTCTTCTTTTGTCCGTGTGCACGTCGGATGGCGAGAGCATAGCACGCGCGCCTTCTGCTAGATGAATCGCAGCCATTTGGAAACTTCGAATCATCCAAACACGTCGATATAACTACTAATTTCCTTGTTTTCTTCATCCTAATATGTGGCGAGCTTAATTTAGTGGTAAAATTGCTGTATAAACTCTGGAGAGAAGTACAATAATTTTGCCTGTTTCGTGCACGAAGATACGCTACCAGCGAAGTGCTTAAATTCAACTCCTCGCCTTATTCAATCCTGCACGAATATTTTATGCGCATACGTACGTGTATAAGTGTATTACGTTAGCTCTGACGAAACCACAAATTAAATATCAACTGTGTTCATCTGGGGGATGTGGCTGCAGGGAGGAAGCGGCACTACAGAGGGGTGAGGCAGCGGCCATGGGGAAAGTGGGCGGCGGAGATCCGGGACCCCAAGAAAGCGGCTCGTGTATGGCTCGGCACCTTTGACACGGCTGAGGACGCCGCCATCGCCTACGACGAAGCGGCGCTGCGCTTCAAGGGCACCAAGGCCAAGCTCAACTTCCCCGAGCGCGTCCAGGGACGCACCGACCTCGGCTTCGTCGTCACGCGCGGCATTCCCGACAGATTGCAGCAACAACAACACTACCCCGCCACCGTGGGGGCGCCGGCAATGCGGCCACCGCCGCACCAGACCGTGGTGCCGTACCCTGACCTCATGCGGTATGCACAGCTGTTGCAGGGCGCTGGCAGTGCCGGGGGCGCTGTCAACCTGCCGTTTGGCGCCATGTCGCCCCCGTCGATGTCCTCGTCGTCGCCGCACATACTCGACTTCTCGACACAGCAGCTCATCCGAGTGAGCCCAGCGTCTCCCGCGGCGGCAATATCGAGCTCAGGCACAACGGGGCCGTCCACCTCATCGTCCACGACCACGGCATCGACGCCAGGTGCTGCATGGCCGTACACTGGGGAGCACAAAAATAATAAAGACTCGTGAGAGATCAGTGGATCGAAGGTGCTTGCGATCCATCGGGACATGTCTTAGCAGTAGATGAGGAATACGCAT

***Hap-2A-2*:**

CCAAATGTTGAGTGACTTGGCGTTGAGATTAGTTCATACAGATCTAAAATATGTCATATAAAAGTGTAATAATTTGAGTATTTGGTGCAAAAAAATTGGTATTATCTCTTTTTCGAATATTTTTTGATTTCTTACACACGTGTCACATAGTAAATTTGGTATTATCTCCATTGAATGAACGCGCAATGTTATTCATACATCATCATATTGTAATAATTTGTCTGTTTAGAGTGTAAAAGATATATTACTTCTTGTCCAGACTCCAAAATGCTGAAATTTTGTACAGTACATATTTTGCACTAAGAAATTTAATGGCAATACCGAAATACTCCGTACAAAGTGCTCAGCCAAAGGTGCTAGAACTGGGACACTGCGCGCCTCTGCCGAGTAACAACAGACTTCTCCTTCTATCACTTCCCCTAGCTATCATTAAAGGCGTTCAGAAGAGCTCGTCTCCTCTCTCTCCCTCCGCGTTCTTATCAGTACGTTGTCCGCGCCTAGGCACCAAAGTCCAAAGCAACAGCCATAGCTCGATCTCGATCCCCGGCGCGACGAAAGAGAAAGAAGCGGCGGCAGGTCGACAGGTCGATCAACTAAGGTGGATCCCCGGAGGCATGGGAAGAGGCCCCTACCCGCCGACGAGGAGGAGGAACAGCCGCCACCGCCGCCGTCAGCAGCCCAGCACGAGCAGGTGGAGGAGCAGCCGTATCACCCCCTCATCGCGCACGCTCTGCAGCAGCAAGGAGCTGCCAGCGCCGGCGGAAGCTCGGGAGCAGATGTGGCCGACCCTTCCCCGTCACCGGAGGCGTACGCGCAGTACTACTACTCGGCGCGCGCCGACCACGACGCCACCGCCATGGTCTCCGCTCTGTCCCACGTCATCCGCGCCACACCGGAACAGCAACAAGCCTACTACCCCGCCGGATCCGCCGCTGTCTCAGGAGAACAGCAGCATCAGCACGATGCGGCGGCTGCCGCGGCCATCGCTGAGGAACAAGGTAATCAACAATAGCTGCTAGCATCCATCGATGTATCCAAACGTAGTCTTCTTTTTTCCGTGTGCACGTCGGATGGCGAGAGCATAGCACGCGCGCCTTCTGCTAGATGAATCGCAGCCATTTGGAAACTTCGAATCATCCAAACACGTCGATATAACTACTAATTTCCTTGTTTTCTTCATCCTAATATGTGGCGAGCTTAATTTAATGGTAAAATTGCTGTATAAACTCTGGAGAGAAGTACAATAATTTTGCCTGTTTCGTGCACGAAGATACGCTACCAGCGAAGTGCTTAAATTCAACTCCTCGCCTTATTCAATCCTGCACGAATATTTTATGCGCATACGTACGTGTATAAGTGTATTACGTTAGCTCTGACGAAACCACAAATTAATTATCAATTGTGTTCATCTGGGGGATGTGGCTGCAGGGAGGAAGCGGCACTACAGAGGGGTGAGGCAGCGGCCATGGGGAAAGTGGGCGGCGGAGATCCGGGACCCCAAGAAAGCGGCTCGTGTATGGCTCGGCACCTTTGACACGGCTGAGGACGCCGCCATCGCCTACGACGAAGCGGCGCTGCGCTTCAAGGGCACCAAGGCCAAGCTCAACTTCCCCGAGCGCGTCCAGGGACGCACCGACCTCGGCTTCGTCGTCACGCGCGGCATTCCCGACAGATTGCAGCAACAACAACACTACCCCGCCACCGTGGGGGCGCCGGCAATGCGGCCACCGCCGCACCAGACCGTGGTGCCGTACCCTGACCTCATGCGGTATGCACAGCTGTTGCAGGGCGCTGGCAGTGCCGGGGGCGCTGTCAACCTGCCGTTTGGCGCCATGTCGCCCCCGTCGATGTCCTCGTCGTCGCCGCACATACTCGACTTCTCGACACAGCAGCTCATCCGAGTGAGCCCAGCGTCTCCCGCGGCGGCAATATCGAGCTCAGGCACAACGGGGCCGTCCACCTCATCGTCCACGACCACGGCATCGTCGCCAGGTGCTGCATGGCCGTACACTGGGGAGCACAAAAATAATAAAGACTCGTGAGAGATCAGTGGATCGAAGGTGCTTGCGATCCATCGGGACATGTCTTAGCAGTAGATGAGGAATACGCAT

***Hap-2A-3*:**

CCAAATGTTGAGTGACTTGGCGTTGAGATTAGTTCATACAGATCTAAAATATGTCATATAAAAGTGTAATAATTTGAGTATTTAGTGCAAAAAAATTGGTATTATCTCTTTTTCGAATATTTTTTGATTTCTTACACACGTGTCACATAGTAAATTTGGTATTATCTCCATTGAATGAACGCGCAATGCTATTCATACATCATCATATTGTAATAATTTGTCTGTTTAGAGTGTAAAAGATATATTACTTCTTGTCCAGACTCCAAAATGCTGAAATTTTGTACAGTACATATTTTGCACTAAGAAATTTAATGGCAATACCGAAATACTCCGTACAAAGTGCTCAGCCAAAGGTGCTAGAACTGGGACACTGCGCGCCTCTGCCGAGTAACAACAGACTTCTCCTTCTATCACTTCCCCTAGCTATCATTAAAGGCGTTCAGAAGAGCTCGTCTCCTCTCTCTCCCTCCGCGTTCTTATCAGTACGTTGTCCGCGCCTAGGCACCAAAGTCCAAAGCAACAGCCATAGCTCGATCTCGATCCCCGGCGCGACGAAAGAGAAAGAAGCGGCGGCAGGTCGACAGGTCGATCAACTAAGGTGGATCCCCGGAGGCATGGGAAGAGGCCCCTACCCGCCGACGAGGAGGAGGAACAGCCGCCACCGCCGCCGTCAGCAGCCCAGCACGAGCAGGTGGAGGAGCAGCCGTATCACCCCCTCATCGCGCGCGCTCTGCAGCAGCAAGGAGCTGCCAGCGCCGGCGGAAGCTCGGGAGCAGATGTGGCCGACCCTTCCCCGTCACCGGAGGCGTACGCGCAGTACTACTACTCGGCGCGCGCCGACCACGACGCCACCGCCATGGTCTCCGCTCTGTCCCACGTCATCCGCGCCACACCGGAACAGCAACAAGCCTACTACCCCGCCGGATCCGCCGCTGTCTCAGGAGAACAGCAGCATCAGCACGATGCGGCGGCTGCCGCGGCCATCGCTGAGGAACAAGGTAATCAACAATAGCTGCTAGCATCCATCGATGTATCCAAACGTAGTCTTCTTTTGTCCGTGTGCACGTCGGATGGCGAGAGCATAGCACGCGCGCCTTCTGCTAGATGAATCGCAGCCATTTGGAAACTTTGAATCATCCAAACACGTCGATATAACTACTAATTTCCTTGTCTTCTTCATCCTAATATGTGGCGAGCTTAATTTAGTGGTAAAATTGCTGTATAAACTCTGGAGAGAAGTACAATAATTTTGCCTGTTTCGTGCACGAAGATACGCTACCAGCGAAGTGCTTAAATTCAACTCCTCGCCTTATTCAATCCTGCACGAATATTTTATGCGCATACGTACGTGTATAAGTGTATTACGTTAGCTCTGACGAAACCACAAATTAATTATCAATTGTGTTCATCTGGGGGATGTGGCTGCAGGGAGGAAGCGGCACTACAGAGGGGTGAGGCAGCGGCCATGGGGAAAGTGGGCGGCGGAGATCCGGGACCCCAAGAAAGCGGCTCGTGTATGGCTCGGCACCTTTGACACGGCTGAGGACGCCGCCATCGCCTACGACGAAGCGGCGCTGCGCTTCAAGGGCACCAAGGCCAAGCTCAACTTCCCCGAGCGCGTCCAGGGACGCACCGACCTCGGCTTCGTCGTCACGCGCGGCATTCCCGACAGATTGCAGCAACAACAACACTACCCCGCCACCGTGGGGGCGCCGGCAATGCGGCCACCGCCGCACCAGACCGTGGTGCCGTACCCTGACCTCATGCGGTATGCACAGCTGTTGCAGGGCGCTGGCAGTGCCGGGGGCGCTGTCAACCTGCCGTTTGGCGCCATGTCGCCCCCGTCGATGTCCTCGTCGTCGCCGCACATACTCGACTTCTCGACACAGCAGCTCATCCGAGTGAGCCCAGCGTCTCCCGCGGCGGCAATATCGAGCTCAGGCACAACGGGGCCGTCCACCTCATCGTCCACGACCACGGCATCGTCGCCAGGTGCTGCATGGCCGTACACTGGGGAGCACAAAAATAATAAAGACCCGTGAGAGATCAGTGGATCGAAGGTGCTTGCGATCCATCGGGACATGTCTTAGCAGTAGATGAGGAATACGCAT

***Hap-2A-4*:**

CCAAATGTTGAGTGACTTGGCGTTGAGATTAGTTCATACAGATCGAAAATATGTCATATAAAAGTGTAATAATTTGAGTATTTAGTGCAAAAAAATTGGTATTATCTCTTTTTCGAATATTTTTTGATTTCTTACACACGTGTCACATAGTAAATTTGGTATTATCTCCATTGAATGAACGCGCAATGCTATTCATACATCATCATATTGTAATAATTTGTCTGTTTAGAGTGTAAAAGATATATTACTTCTTGTCCAGACTCCAAAATGCTGAAATTTTGTACAGTACATATTTTGCACTAAGAAATTTAATGGCAATGCCGAAATACTCCGTACAAAGTGCTCAGCCAAAGGTGCTAGAACTGGGACACTGCGCGCCTCTGCCGAGTAACAACAGACTTCTCCTTCTATCACTTCCCTAGCTATCATTAAAGGCGTTCAGAAGAGCTCGTCTCCTCTCTCTCCCTCCGCGTTCTTATCAGTACGTTGTCCGCGCCTAGGCACCAAAGTCCAAAGCAACAGCCATAGCTCGATCTCGATCCCCGGCGCGACGAAAGAGAAAGAAGCGGCGGCAGGTCGACAGGTCGATCAACTAAGGTGGATCCCCGGAGGCATGGGAAGAGGCCCCTACCCGCCGACGAGGAGGAGGAACAGCCGCCACCGCCGCCGTCAGCAGCCAAGCACGAGCAGGTGGAGGAGCAGCCGTATCACCCCCTCATCGCGCACGCTCTGCAGCAGCAAGGAGCTGCCAGCGCCGGCGGAAGCTCGGGAGCAGATGTGGCCGACCCTTCCCCGTCACCGGAGGCGTACGCGCAGTACTACTACTCGGCGCGCGCCGACCACGACGCCACCGCCATGGTCTCCGCTCTGTCCCACGTCATCCGCGCCACACCGGAACAGCAACAAGCCTATTACCCCGCCGGATCCGCCGCTGTCTCAGGAGAACAGCAGCATCAGCACGATGCGGCGGCTGCCGCGGCCATCGTTGAGGAACAAGGTAATCAACAATAGCTGCTAGCATCCATCGATGTATCCAAACGTAGTCTTCTTTTGTCCGTGTGCACGTCGGATGGCGAGAGCATAGCACGCGCGCCTTCTGCTAGATGAATCGCAGCCATTTGGAAACTTCGAATCATCCAAACACGTCGATATAACTACTAATTTCCTTGTTTTCTTCATCCTAATATGTGGCGAGCTTAATTTAGTGGTAAAATTGCTGTATAAACTCTGGAGAGAAGTACAATAATTTTGCCTGTTTCGTGCACGAAGATACGCTACCAGCGAAGTGCTTAAATTCAACTCCTCGCCTTATTCAATCCTGCACGAATATTTTATGCGCATACGTACGTGTATAAGTGTATTACGTTAGCTCTGACGAAACCACAAATTAATTATCAATTGTGTTCATCTGGGGGATGTGGCTGCAGGGAGGAAGCGGCACTACAGAGGGGTGAGGCAGCGGCCATGGGGAAAGTGGGCGGCGGAGATCCGGGACCCCAAGAAAGCGGCTCGTGTATGGCTCGGCACCTTTGACACGGCTGAGGACGCCGCCATCGCCTACGACGAAGCGGCGCTGCGCTTCAAGGGCACCAAGGCCAAGCTCAACTTCCCCGAGCGCGTCCAGGGACGCACCGACCTCGGCTTCGTCGTCACGCGCGGCATTCCCGACAGATTGCAGCAACAACAACACTACCCCGCCACCGTGGGGGCGCCGGCAATGCGGCCACCGCCGCACCAGACCGTGGTGCCGTACCCTGACCTCATGCGGTATGCACAGCTGTTGCAGGGCGCTGGCAGTGCCGGGGGCGCTGTCAACCTGCCGTTTGGCGCCATGTCGCCCCCGTCGATGTCCTCGTCGTCGCCGCACATACTCGACTTCTCGACACAACAGCTCATCCGAGTGAGCCCAGCGTCTCCCGCGGCGGCAATATCGAGCTCAGGCACAACGGGGCCGTCCACCTCATCGTCCACGACCACGGCATCGTCGCCAGGTGCTGCATGGCCGTACACTGGGGAGCACAAAAATAATAAAGACTCGTGAGAGATCAGTGGATCGAAGGTGCTTGCGATCCATCGGGACATGTCTTAGCAGTAGATGAGGAATACGCAT

***Hap-2A-5*:**

CCAAATGTTGAGTGACTTGGCGTTGAGATTAGTTCATACAGATCGAAAATATGTCATATAAAAGTGTAATAATTTGAGTATTTAGTGCAAAAAAATTGGTATTATCTCTTTTTCGAATATTTTTTGATTTCTTACACACGTGTCACATAGTAAATTTGGTATTATCTCCATTGAATGAACGCGCAATGTTATTCATACATCATCATATTGTAATAATTTGTCTGTTTAGAGTGTAAAAGATATATTACTTCTTGTCCAGACTCCAAAATGCTGAAATTTTGTACAGTACATATTTTGCACTAAGAAATTTAATGGCAATGCCGAAATACTCCGTACAAAGTGCTCAGCCAAAGGTGCTAGAACTGGGACACTGCGCGCCTCTGCCGAGTAACAACAGACTTCTCCTTCTATCACTTCCCTAGCTATCATTAAAGGCGTTCAGAAGAGCTCGTCTCCTCTCTCTCCCTCCGCGTTCTTATCAGTACGTTGTCCGCGCCTAGGCACCAAAGTCCAAAGCAACAGCCATAGCTCGATCTCGATCCCCGGCGCGACGAAAGAGAAAGAAGCGGCGGCAGGTCGACAGGTCGATCAACTAAGGTGGATCCCCGGAGGCATGGGAAGAGGCCCCTACCCGCCGACGAGGAGGAGGAACAGCCGCCACCGCCGCCGTCAGCAGCCAAGCACGAGCAGGTGGAGGAGCAGCCGTATCACCCCCTCATCGCGCACGCTCTGCAGCAGCAAGGAGCTGCCAGCGCCGGCGGAAGCTCGGGAGCAGATGTGGCCGACCCTTCCCCGTCACCGGAGGCGTACGCGCAGTACTACTACTCGGCGCGCGCCGACCACGACGCCACCGCCATGGTCTCCGCTCTGTCCCACGTCATCCGCGCCACACCGGAACAGCAACAAGCCTATTACCCCGCCGGATCCGCCGCTGTCTCAGGAGAACAGCAGCATCAGCACGATGCGGCGGCTGCCGCGGCCATCGTTGAGGAACAAGGTAATCAACAATAGCTGCTAGCATCCATCGATGTATCCAAACGTAGTCTTCTTTTGTCCGTGTGCACGTCGGATGGCGAGAGCATAGCACGCGCGCCTTCTGCTAGATGAATCGCAGCCATTTGGAAACTTCGAATCATCCAAACACGTCGATATAACTACTAATTTCCTTGTTTTCTTCATCCTAATATGTGGCGAGCTTAATTTAGTGGTAAAATTGCTGTATAAACTCTGGAGAGAAGTACAATAATTTTGCCTGTTTCGTGCACGAAGATACGCTACCAGCGAAGTGCTTAAATTCAACTCCTCGCCTTATTCAATCCTGCACGAATATTTTATGCGCATACGTACGTGTATAAGTGTATTACGTTAGCTCTGACGAAACCACAAATTAATTATCAATTGTGTTCATCTGGGGGATGTGGCTGCAGGGAGGAAGCGGCACTACAGAGGGGTGAGGCAGCGGCCATGGGGAAAGTGGGCGGCGGAGATCCGGGACCCCAAGAAAGCGGCTCGTGTATGGCTCGGCACCTTTGACACGGCTGAGGACGCCGCCATCGCCTACGACGAAGCGGCGCTGCGCTTCAAGGGCACCAAGGCCAAGCTCAACTTCCCCGAGCGCGTCCAGGGACGCACCGACCTCGGCTTCGTCGTCACGCGCGGCATTCCCGACAGATTGCAGCAACAACAACACTACCCCGCCACCGTGGGGGCGCCGGCAATGCGGCCACCGCCGCACCAGACCGTGGTGCCGTACCCTGACCTCATGCGGTATGCACAGCTGTTGCAGGGCGCTGGCAGTGCCGGGGGCGCTGTCAACCTGCCGTTTGGCGCCATGTCGCCCCCGTCGATGTCCTCGTCGTCGCCGCACATACTCGACTTCTCGACACAACAGCTCATCCGAGTGAGCCCAGCGTCTCCCGCGGCGGCAATATCGAGCTCAGGCACAACGGGGCCGTCCACCTCATCGTCCACGACCACGGCATCGTCGCCAGGTGCTGCATGGCCGTACACTGGGGAGCACAAAAATAATAAAGACTCGTGAGAGATCAGTGGATCGAAGGTGCTTGCGATCCATCGGGACATGTCTTAGCAGTAGATGAGGAATACGCAT

***Hap-2A-6*:**

CCAAATGTTGAGTGACTTGGCGTTGAGATTAGTTCATACAGATCGAAAATATGTCATATAAAAGTGTAATAATTTGAGTATTTAGTGCAAAAAAATTGGTATTATCTCTTTTTCGAATATTTTTTGATTTCTTACACACGTGTCACATAGTAAATTTGGTATTATCTCCATTGAATGAACGCGCAATGCTATTCATACATCATCATATTGTAATAATTTGTCTGTTTAGAGTGTAAAAGATATATTACTTCTTGTCCAGACTCCAAAATGCTGAAATTTTGTACAGTACATATTTTGCACTAAGAAATTTAATGGCAATGCCGAAATACTCCGTACAAAGTGCTCAGCCAAAGGTGCTAGAACTGGGACACTGCGCGCCTCTGCCGAGTAACAACAGACTTCTCCTTCTATCACTTCCCTAGCTATCATTAAAGGCGTTCAGAAGAGCTCGTCTCCTCTCTCTCCCTCCGCGTTCTTATCAGTACGTTGTCCGCGCCTAGGCACCAAAGTCCAAAGCAACAGCCATAGCTCGATCTCGATCCCCGGCGCGACGAAAGAGAAAGAAGCGGCGGCAGGTCGACAGGTCGATCAACTAAGGTGGATCCCCGGAGGCATGGGAAGAGGCCCCTACCCGCCGACGAGGAGGAGGAACAGCCGCCACCGCCGCCGTCAGCAGCCAAGCACGAGCAGGTGGAGGAGCAGCCGTATCACCCCCTCATCGCGCACGCTCTGCAGCAGCAAGGAGCTGCCAGCGCCGGCGGAAGCTCGGGAGCAGATGTGGCCGACCCTTCCCCGTCACCGGAGGCGTACGCGCAGTACTACTACTCGGCGCGCGCCGACCACGACGCCACCGCCATGGTCTCCGCTCTGTCCCACGTCATCCGCGCCACACCGGAACAGCAACAAGCCTATTACCCCGCCGGATCCGCCGCTGTCTCAGGAGAACAGCAGCATCAGCACGATGCGGCGGCTGCCGCGGCCATCGTTGAGGAACAAGGTAATCAGCAATAGCTGCTAGCATCCATCGATGTATCCAAACGTAGTCTTCTTTTGTCCGTGTGCACGTCGGATGGCGAGAGCATAGCACGCGCGCCTTCTGCTAGATGAATCGCAGCCATTTGGAAACTTCGAATCATCCAAACACGTCGATATAACTACTAATTTCCTTGTTTTCTTCATCCTAATATGTGGCGAGCTTAATTTAGTGGTAAAATTGCTGTATAAACTCTGGAGAGAAGTACAATAATTTTGCCTGTTTCGTGCACGAAGATACGCTACCAGCGAAGTGCTTAAATTCAACTCCTCGCCTTATTCAATCCTGCACGAATATTTTATGCGCATACGTACGTGTATAAGTGTATTACGTTAGCTCTGACGAAACCACAAATTAATTATCAATTGTGTTCATCTGGGGGATGTGGCTGCAGGGAGGAAGCGGCACTACAGAGGGGTGAGGCAGCGGCCATGGGGAAAGTGGGCGGCGGAGATCCGGGACCCCAAGAAAGCGGCTCGTGTATGGCTCGGCACCTTTGACACGGCTGAGGACGCCGCCATCGCCTACGACGAAGCGGCGCTGCGCTTCAAGGGCACCAAGGCCAAGCTCAACTTCCCCGAGCGCGTCCAGGGACGCACCGACCTCGGCTTCGTCGTCACGCGCGGCATTCCCGACAGATTGCAGCAACAACAACACTACCCCGCCACCGTGGGGGCGCCGGCAATGCGGCCACCGCCGCACCAGACCGTGGTGCCGTACCCTGACCTCATGCGGTATGCACAGCTGTTGCAGGGCGCTGGCAGTGCCGGGGGCGCTGTCAACCTGCCGTTTGGCGCCATGTCGCCCCCGTCGATGTCCTCGTCGTCGCCGCACATACTCGACTTCTCGACACAACAGCTCATCCGAGTGAGCCCAGCGTCTCCCGCGGCGGCAATATCGAGCTCAGGCACAACGGGGCCGTCCACCTCATCGTCCACGACCACGGCATCGTCGCCAGGTGCTGCATGGCCGTACACTGGGGAGCACAAAAATAATAAAGACTCGTGAGAGATCAGTGGATCGAAGGTGCTTGCGATCCATCGGGACATGTCTTAGCAGTAGATGAGGAATACGCAT

***Hap-2A-7*:**

CCAAATGTTGAGTGACTTGGCGTTGAGATTAGTTCATACAGATCTAAAATATGTCATATAAAAGTGTAATAATTTGAGTATTTAGTGCAAAAAAATTGGTATTATCTCTTTTTCGAATATTTTTTGATTTCTTACACACGTGTCACATAGTAAATTTGGTATTATCTCCATTGAATGAACGCGCAATGTTATTCATACATCATCATATTGTAATAATTTGTCTGTTTAGAGTGTAAAAGATATATTACTTCTTGTCCAGACTCCAAAATGCTGAAATTTTGTACAGTACATATTTTGCACTAAGAAATTTAATGGCAATACCGAAATACTCCGTACAAAGTGCTCAGCCAAAGGTGCTAGAACTGGGACACTGCGCGCCTCTGCCGAGTAACAACAGACTTCTCCTTCTATCACTTCCCCTAGCTATCATTAAAGGCGTTCAGAAGAGCTCGTCTCCTCTCTCTCCCTCCGCGTTCTTATCAGTACGTTGTCCGCGCCTAGGCACCAAAGTCCAAAGCAACAGCCATAGCTCGATCTCGATCCCCGGCGCGACGAAAGAGAAAGAAGCGGCGGCAGGTCGACAGGTCGATCAACTAAGGTGGATCCCCGGAGGCATGGGAAGAGGCCCCTACCCGCCGACGAGGAGGAGGAACAGCCGCCACCGCCGCCGTCAGCAGCCCAGCACGAGCAGGTGGAGGAGCAGCCGTATCACCCCCTCATCGCGCGCGCTCTGCAGCAGCAAGGAGCTGCCAGCGCCGGCAGAAGCTCGGGAGCAGATGTGGCCGACCCTTCCCCGTCACCGGAGGCGTACGCGCAGTACTACTACTCGGCGCGCGCCGACCACGACGCCACCGCCATGGTCTCCGCTCTGTCCCACGTCATCCGCGCCACACCGGAACAGCAACAAGCCTACTACCCCGCCGGATCCGCCGCTGTCTCAGGAGAACAGCAGCATCAGCACGATGCGGCGGCTGCCGCGGCCATCGCTGAGGAACAAGGTAATCAACAATAGCTGCTAGCATCCATCGATGTATCCAAACGTAGTCTTCTTTTGTCCGTGTGCACGTCGGATGGCGAGAGCATAGCACGCGCGCCTTCTGCTAGATGAATCGCAGCCATTTGGAAACTTCGAATCATCCAAACACGTCGATATAACTACTAATTTCCTTGTCTTCTTCATCCTAATATGTGGCGAGCTTAATTTAGGTAAAATTGCTGTATAAACTCTGGAGAGAAGTACAATAATTTTGCCTGTTTCGTGCACGAAGATACGCTACCAGCGAAGTGCTTAAATTCAACTCCTCGCCTTATTCAATCCTGCACGAATATTTTATGCGCATACGTACGTGTATAAGTGTATTACGTTAGCTCTGACGAAACCACAAATTAATTATCAATTGTGTTCATCTGGGGGATGTGGCTGCAGGGAGGAAGCGGCACTACAGAGGGGTGAGGCAGCGGCCATGGGGAAAGTGGGCGGCGGAGATCCGGGACCCCAAGAAAGCGGCTCGTGTATGGCTCGGCACCTTTGACACGGCTGAGGACGCCGCCATCGCCTACGACGAAGCGGCGCTGCGCTTCAAGGGCACCAAGGCCAAGCTCAACTTCCCCGAGCGCGTCCAGGGACGCACCGACCTCGGCTTCGTCGTCACGCGCGGCATTCCCGACAGATTGCAGCAACAACAACACTACCCCGCTACCGTGGGGGCGCCGGCAATGCGGCCACCGCCGCACCAGACCGTGGTGCCGTACCCTGACCTCATGCGGTATGCACAGCTGTTGCAGGGCGCTGGCAGTGCCGGGGGCGCTGTCAACCTGCCGTTTGGCGCCATGTCGCCCCCGTCGATGTCCTCGTCGTCGCCGCACATACTCGACTTCTCGACACAGCAGCTCATCCGAGTGAGCCCAGCGTCTCCCGCGGCGGCAATATCGAGCTCAGGCACAACGGGGCCGTCCACCTCATCGTCCACGACCACGGCATCGTCGCCAGGTGCTGCATGGCCGTACACTGGGGAGCACAAAAATAATAAAGACCCGTGAGAGATCAGTGGATCGAAGGTGCTTGCGATCCATCGGGACATGTCTTAGCAGTAGATGAGGAATACGCAT

***Hap-2A-8*:**

CCAAATGTTGAGTGACTTGGCGTTGAGATTAGTTCATACAGATCTAAAATATGTCATATAAAAGTGTAATAATTTGAGTATTTAGTGCAAAAAAATTGGTATTATCTCTTTTTCGAATATTTTTTGATTTCTTACACACGTGTCACATAGTAAATTTGGTATTATCTCCATTGAATGAACGCGCAATGTTATTCATACATCATCATATTGTAATAATTTGTCTGTTTAGAGTGTAAAAGATATATTACTTCTTGTCCAGACTCCAAAATGCTGAAATTTTGTACAGTACATATTTTGCACTAAGAAATTTAATGGCAATACCGAAATACTCCGTACAAAGTGCTCAGCCAAAGGTGCTAGAACTGGGACACTGCGCGCCTCTGCCGAGTAACAACAGACTTCTCCTTCTATCACTTCCCCTAGCTATCATTAAAGGCGTTCAGAAGAGCTCGTCTCCTCTCTCTCCCTCCGCGTTCTTATCAGTACGTTGTCCGCGCCTAGGCACCAAAGTCCAAAGCAACAGCCATAGCTCGATCTCGATCCCCGGCGCGACGAAAGAGAAAGAAGCGGCGGCAGGTCGACAGGTCGATCAACTAAGGTGGATCCCCGGAGGCATGGGAAGAGGCCCCTACCCGCCGACGAGGAGGAGGAACAGCCGCCACCGCCGCCGTCAGCAGCCCAGCACGAGCAGGTGGAGGAGCAGCCGTATCACCCCCTCATCGCGCGCGCTCTGCAGCAGCAAGGAGCTGCCAGCGCCGGCGGAAGCTCGGGAGCAGATGTGGCCGACCCTTCCCCGTCACCGGAGGCGTACGCGCAGTACTACTACTCGGCGCGCGCCGACCACGACGCCACCGCCATGGTCTCCGCTCTGTCCCACGTCATCCGCGCCACACCGGAACAGCAACAAGCCTACTACCCCGCCGGATCCGCCGCTGTCTCAGGAGAACAGCAGCATCAGCACGATGCGGCGGCTGCCGCGGCCATCGCTGAGGAACAAGGTAATCAACAATAGCTGCTAGCATCCATCGATGTATCCAAACGTAGTCTTCTTTTGTCCGTGTGCACGTCGGATGGCGAGAGCATAGCACGCGCGCCTTCTGCTAGATGAATCGCAGCCATTTGGAAACTTTGAATCATCCAAACACGTCGATATAACTACTAATTTCCTTGTCTTCTTCATCCTAATATGTGGCGAGCTTAATTTAGTGGTAAAATTGCTGTATAAACTCTGGAGAGAAGTACAATAATTTTGCCTGTTTCGTGCACGAAGATACGCTACCAGCGAAGTGCTTAAATTCAACTCCTCGCCTTATTCAATCCTGCACGAATATTTTATGCGCATACGTACGTGTATAAGTGTATTACGTTAGCTCTGACGAAACCACAAATTAATTATCAATTGTGTTCATCTGGGGGATGTGGCTGCAGGGAGGAAGCGGCACTACAGAGGGGTGAGGCAGCGGCCATGGGGAAAGTGGGCGGCGGAGATCCGGGACCCCAAGAAAGCGGCTCGTGTATGGCTCGGCACCTTTGACACGGCTGAGGACGCCGCCATCGCCTACGACGAAGCGGCGCTGCGCTTCAAGGGCACCAAGGCCAAGCTCAACTTCCCCGAGCGCGTCCAGGGACGCACCGACCTCGGCTTCGTCGTCACGCGCGGCATTCCCGACAGATTGCAGCAACAACAACACTACCCCGCCACCGTGGGGGCGCCGGCAATGCGGCCACCGCCGCACCAGACCGTGGTGCCGTACCCTGACCTCATGCGGTATGCACAGCTGTTGCAGGGCGCTGGCAGTGCCGGGGGCGCTGTCAACCTGCCGTTTGGCGCCATGTCGCCCCCGTCGATGTCCTCGTCGTCGCCGCACATACTCGACTTCTCGACACAGCAGCTCATCCGAGTGAGCCCAGCGTCTCCCGCGGCGGCAATATCGAGCTCAGGCACAACGGGGCCGTCCACCTCATCGTCCACGACCACGGCATCGTCGCCAGGTGCTGCATGGCCGTACACTGGGGAGCACAAAAATAATAAAGACCCGTGAGAGATCAGTGGATCGAAGGTGCTTGCGATCCATCGGGACATGTCTTAGCAGTAGATGAGGAATACGCAT

***Hap-2A-9*:**

CCAAATGTTGAGTGACTTGGCGTTGAGATTAGTTCATACAGATCTAAAATATGTCATATAAAAGTGTAATAATTTGAGTATTTAGTGCAAAAAAATTGGTATTATCTCTTTTTCGAATATTTTTTGATTTCTTACACACGTGTCACATAGTAAATTTGGTATTATCTCCATTGAATGAACGCGCAATGTTATTCATACATCATCATATTGTAATAATTTGTCTGTTTAGAGTGTAAAAGATATATTACTTCTTGTCCAGACTCCAAAATGCTGAAATTTTGTACAGTACATATTTTGCACTAAGAAATTTAATGGCAATACCGAAATACTCCGTACAAAGTGCTCAGCCAAAGGTGCTAGAACTGGGACACTGCGCGCCTCTGCCGAGTAACAACAGACTTCTCCTTCTATCACTTCCCCTAGCTATCATTAAAGGCGTTCAGAAGAGCTCGTCTCCTCTCTCTCCCTCCGCGTTCTTATCAGTACGTTGTCCGCGCCTAGGCACCAAAGTCCAAAGCAACAGCCATAGCTCGATCTCGATCCCCGGCGCGACGAAAGAGAAAGAAGCGGCGGCAGGTCGACAGGTCGATCAACTAAGGTGGATCCCCGGAGGCATGGGAAGAGGCCCCTACCCGCCGACGAGGAGGAGGAACAGCCGCCACCGCCGCCGTCAGCAGCCCAGCACGAGCAGGTGGAGGAGCAGCCGTATCACCCCCTCATCGCGCGCGCTCTGCAGCAGCAAGGAGCTGCCAGCGCCGGCGGAAGCTCGGGAGCAGATGTGGCCGACCCTTCCCCGTCACCGGAGGCGTACGCGCAGTACTACTACTCGGCGCGCGCCGACCACGACGCCACCGCCATGGTCTCCGCTCTGTCCCACGTCATCCGCGCCACACCGGAACAGCAACAAGCCTACTACCCCGCCGGATCCGCCGCTGTCTCAGGAGAACAGCAGCATCAGCACGATGCGGCGGCTGCCGCGGCCATCGCTGAGGAACAAGGTAATCAACAATAGCTGCTAGCATCCATCGATGTATCCAAACGTAGTCTTCTTTTGTCCGTGTGCACGTCGGATGGCGAGAGCATAGCACGCGCGCCTTCTGCTAGATGAATCGCAGCCATTTGGAAACTTCGAATCATCCAAACACGTCGATATAACTACTAATTTCCTTGTCTTCTTCATCCTAATATGTGGCGAGCTTAATTTAGTGGTAAAATTGCTGTATAAACTCTGGAGAGAAGTACAATAATTTTGCCTGTTTCGTGCACGAAGATACGCTACCAGCGAAGTGCTTAAATTCAACTCCTCGCCTTATTCAATCCTGCACGAATATTTTATGCGCATACGTACGTGTATAAGTGTATTACGTTAGCTCTGACGAAACCACAAATTAATTATCAATTGTGTTCATCTGGGGGATGTGGCTGCAGGGAGGAAGCGGCACTACAGAGGGGTGAGGCAGCGGCCATGGGGAAAGTGGGCGGCGGAGATCCGGGACCCCAAGAAAGCGGCTCGTGTATGGCTCGGCACCTTTGACACGGCTGAGGACGCCGCCATCGCCTACGACGAAGCGGCGCTGCGCTTCAAGGGCACCAAGGCCAAGCTCAACTTCCCCGAGCGCGTCCAGGGACGCACCGACCTCGGCTTCGTCGTCACGCGCGGCATTCCCGACAGATTGCAGCAACAACAACACTACCCCGCCACCGTGGGGGCGCCGGCAATGCGGCCACCGCCGCACCAGACCGTGGTGCCGTACCCTGACCTCATGCGGTATGCACAGCTGTTGCAGGGCGCTGGCAGTGCCGGGGGCGCTGTCAACCTGCCGTTTGGCGCCATGTCGCCCCCGTCGATGTCCTCGTCGTCGCCGCACATACTCGACTTCTCGACACAGCAGCTCATCCGAGTGAGCCCAGCGTCTCCCGCGGCGGCAATATCGAGCTCAGGCACAACGGGGCCGTCCACCTCATCGTCCACGACCACGGCATCGTCGCCAGGTGCTGCATGGCCGTACACTGGGGAGCACAAAAATAATAAAGACCCGTGAGAGATCAGTGGATCGAAGGTGCTTGCGATCCATCGGGACATGTCTTAGCAGTAGATGAGGAATACGCAT

***Hap-2A-10*:**

CCAAATGTTGAGTGACTTGGCGTTGAGATTAGTTCATACAGATCTAAAATATGTCATATAAAAGTGTAATAATTTGAGTATTTAGTGCAAAAAAATTGGTATTATCTCTTTTTCGAATATTTTTTGATTTCTTACACACGTGTCACATAGTAAATTTGGTATTATCTCCATTGAATGAAGGCGCAATGTTATTCATACATCATCATATTGTAATAATTTGTCTGTTTAGAGTGTAAAAGATATATTACTTCTTGTCCAGACTCCAAAATGCTGAAATTTTGTACAGTACATATTTTGCACTAAGAAATTTAATGGCAATACCGAAATACTCCGTACAAAGTGCTCAGCCAAAGGTGCTAGAACTGGGACACTGCGCGCCTCTGCCGAGTAACAACAGACTTCTCCTTCTATCACTTCCCCTAGCTATCATTAAAGGCGTTCAGAAGAGCTCGTCTCCTCTCTCTCCCTCCGCGTTCTTATCAGTACGTTGTCCGCGCCTAGGCACCAAAGTCCAAAGCAACAGCCATAGCTCGATCTCGATCCCCGGCGCGACGAAAGAGAAAGAAGCGGCGGCAGGTCGACAGGTCGATCAACTAAGGTGGATCCCCGGAGGCATGGGAAGAGGCCCCTACCCGCCGACGAGGAGGAGGAACAGCCGCCACCGCCGCCGTCAGCAGCCCAGCACGAGCAGGTGGAGGAGCAGCCGTATCACCCCCTCATCGCGCGCGCTCTGCAGCAGCAAGGAGCTGCCAGCGCCGGCGGAAGCTCGGGAGCAGATGTGGCCGACCCTTCCCCGTCACCGGAGGCGTACGCGCAGTACTACTACTCGGCGCGCGCCGACCACGACGCCACCGCCATGGTCTCCGCTCTGTCCCACGTCATCCGCGCCACACCGGAACAGCAACAAGCCTACTACCCCGCCGGATCCGCCGCTGTCTCAGGAGAACAGCAGCATCAGCACGATGCGGCGGCTGCCGCGGCCATCGCTGAGGAACAAGGTAATCAACAATAGCTGCTAGCATCCATCGATGTATCCAAACGTAGTCTTCTTTTGTCCGTGTGCACGTCGGATGGCGAGAGCATAGCACGCGCGCCTTCTGCTAGATGAATCGCAGCCATTTGGAAACTTCGAATCATCCAAACACGTCGATATAACTACTAATTTCCTTGTCTTCTTCATCCTAATATGTGGCGAGCTTAATTTAGTGGTAAAATTGCTGTATAAACTCTGGAGAGAAGTACAATAATTTTGCCTGTTTCGTGCACGAAGATACGCTACCAGCGAAGTGCTTAAATTCAACTCCTCGCCTTATTCAATCCTGCACGAATATTTTATGCGCATACGTACGTGTATAAGTGTATTACGTTAGCTCTGACGAAACCACAAATTAATTATCAATTGTGTTCATCTGGGGGATGTGGCTGCAGGGAGGAAGCGGCACTACAGAGGGGTGAGGCAGCGGCCATGGGGAAAGTGGGCGGCGGAGATCCGGGACCCCAAGAAAGCGGCTCGTGTATGGCTCGGCACCTTTGACACGGCTGAGGACGCCGCCATCGCCTACGACGAAGCGGCGCTGCGCTTCAAGGGCACCAAGGCCAAGCTCAACTTCCCCGAGCGCGTCCAGGGACGCACCGACCTCGGCTTCGTCGTCACGCGCGGCATTCCCGACAGATTGCAGCAACAACAACACTACCCCGCCACCGTGGGGGCGCCGGCAATGCGGCCACCGCCGCACCAGACCGTGGTGCCGTACCCTGACCTCATGCGGTATGCACAGCTGTTGCAGGGCGCTGGCAGTGCCGGGGGCGCTGTCAACCTGCCGTTTGGCGCCATGTCGCCCCCGTCGATGTCCTCGTCGTCGCCGCACATACTCGACTTCTCGACACAGCAGCTCATCCGAGTGAGCCCAGCGTCTCCCGCGGCGGCAATATCGAGCTCAGGCACAACGGGGCCGTCCACCTCATCGTCCACGACCACGGCATCGTCGCCAGGTGCTGCATGGCCGTACACTGGGGAGCACAAAAATAATAAAGACCCGTGAGAGATCAGTGGATCGAAGGTGCTTGCGATCCATCGGGACATGTCTTAGCAGTAGATGAGGAATACGCAT

***Hap-2B-1*:**

GAATTGCCCTTGAATGAACGGGAAATGTTATCCATACATCATCATATTGTAATAATTCGTCTGTTTAATTAGAGTGTAAAAGATATATTACTCCTTGTTAGACTCCAAAATGCTGAAATTTTGTACATATTTTGCACTAAGAAATTTAATGGCAATACCGAAATACGCCGTACAAAGTGCTCAGCCAAAGGTGCTAGAACTGGGACACTGCGCGCCTCTGCCGAGTAACAACAGACTTCTTCTATCACTTCCCCTAGCTATCATTAAAAGCGTTCAGAAGAGCTCGTCTCCTCTCTCTCCCTCCGCGTTCTTATCAGTACGTTGTCCGCGCCTAGGCACCAAAGTCCAAAGCAACAGCCATAGCTCGATCTCGATCCCCGGCGCGACGAAAGAAAAAGAAGCGGCGGCAGGTCGACAGGTCGATCAACTAAGGTGGATCCCCGGAGGCATGGGAAGAGGCCCCTACCCGCCGACGAGGAGGAGGAACAGCCGCCACCGCCGCCGTCAGCAGCCAAGCACGAGCAGGTGGAGGAGCAGCCGTATCACCACCTCATCGGGCGCGCTCTGCAGCAGCAAGGAGCTGCCAGCGCCTGCGGAAGCTCGGGAGCAGATGTGGCCGACCCTTCCCCGTCACCGGAGGCGTACGCGCAGTACTACTACTCGGCGCGCGCCGACCACGACGCCACCGCCATGGTCTCCGCTCTGTCCCACGTCATCCGCGCCACACCGGACCAGCAACAAGCCTACTACCCCGCCGGATCCGCCGCTGTCTCAGGAGAACAGCAGCATCAGCACGATGCGGCGGCTGCCGCGGCCATCGCTGAGGAACAAGGTAATCAACAATAGCTACTAGCATCCATCGATGTATCCAAACGTAGTCTTCTTTTGTCCGTGTGCACGTCGGATGGCGAGAGCATAGCACGCGCGCCTTCTGCTAGATGAATCGCAGCCATTTGGAAACTTCGAATCATCCAAACACGTCGATATAACTACTAATTTCCTTGTTTTCTTCATCCTAATATGTGGCGAGCTTAATTTAGTGGTAAAATGGCTGTATAAACTCTGGAGAGAAGTACAATAATTTTGCCTGTTTCGTGCACGAAGATACGCTAGCAGCGAAGTGCTTAAATTCAACTCCTCTCCTTATTCAATCCTGCACGAATATTTTATGCGCATACGTACGTGTATAAGTGTATTACGTTAGCTCTGACGAAACCACAAATTAATTATCAATTGTGTTCATCTGGGGGATGTGGCTGCAGGGAGGAAGCGGCACTACAGAGGGGTGAGGCAGCGGCCATGGGGAAAGTGGGCGGCGGAGATCCGGGACCCCAAGAAAGCGGCTCGTGTGTGGCTCGGCACCTTTGACACGGCTGAGGACGCCGCCATCGCCTACGACGAAGCGGCGCTGCGCTTCAAGGGCACCAAGGCCAAGCTCAACTTCCCCGAGCGCGTCCAGGGACGCACCGACCTCGGCTTCGTTGTCACGCGCGGCATACCCGACAGATTGCAGCAACAACAACACTACCCCGCCACCGTGGGGGCGCCGGCAATGCGGCCACCGCCGCACCAGCTGCAGACCGTGGTGCCGTACCCTGACCTCATGCGGTATGCACGGCTGTTGCAGGGCGCTGGCAGTGCCGGGGGCGCTGTCAACCTGCCGTTCGGCGCCATGTCGCCCCCGTCGATGTCCTCGTCGGCGCCGCACATACTCGACTTCTCGACACAGCAGCTCATCCGAGTGAGCCCGGCGTCTCCCGCGGCGGCAATGTCGAGCTCAGGCACAACGGGGCCGTCCACCTCATCGTCCACGACTACGGCATCGTCGCCAGGTGCTGCATGGCCGTACACTGGGGAGCACAAAAATAATAAAGACTCGTGAGAGATCAATGGATCGAAGGTGCTTGCGATCCATCGGGACATGTCTTAGCAGTAGATGAGGAATACGCATGCATTCATCATGCAATAAGGTGGGTAAGTGTTGGTCAATTAATTTTGCTGGTGAACATTTCTTTCCTTCTTCTTATCCCCAAATTTGTTTATCTAAAGCTCTTGTTTTGTTTATTTTTACTTTAATAATGGGTGATTTTTTTTGGCGGGTAATGGGTGACTCTTAACTAGCTAGGTACTAGTTGTACTAAATTAGTGACAAGTAACATAGAACAGAAGGAGACCTTGTTCCTTGGTCTAATCGTTTATCGTTTC

***Hap-2B-2*:**

GGGCCCTCTAGATGCATGCTCGAGCGGCCGCCAGTGTGATGGATATCTGCAGAATTGCCCTTGAATGAACGGGAAATGTTATCCATACATCATCATATTGTAATAATTCGTCTGTTTAATTAGAGTGTAAAAGATATATTACTCCTTGTTAGACTCCAAAATGCTGAAATTTTGTACATATTTTGCACTAAGAAATTTAATGGCAATACCGAAATACGCCGTACAAAGTGCTCAGCCAAAGGTGCTAGAACTGGGACACTGCGCGCCTCTGCCGAGTAACAACAGACTTCTCCTTCTATCACTTCCCCTAGCTATCATTAAAAGCGTTCAGAAGAGCTCGTCTCCTCTCTCTCCCTCCGCGTTCTTATCAGTACGTTGTCCGCGCCTAGGCACCAAAGTCCAAAGCAACAGCCATAGCTCGATCTCGATCCCCGGCGCGACGAAAGAAAAAGAAGCGGCGGCAGGTCGACAGGTCGATCAACTAAGGTGGATCCCCGGAGGCATGGGAaGAGGCCCCTACCCGCCGACGAGGAGGAGGAACAGCCGCCACCGCCGCCGTCAGCAGCCAAGCACGAGCAGGTGGAGGAGCAGCCGTATCACCACCTCATCGGGCGCGCTCTGCAGCAGCAAGGAGCTGCCAGCGCCGGCGGAAGCTCGGGAGCAGATGTGGCCGACCCTTCCCCGTCACCGGAGGCGTACGCGCAGTACTACTACTCGGCGCGCGCCGACCACGACGCCACCGCCATGGTCTCCGCTCTGTCCCACGTCATCCGCGCCACACCGGACCAGCAACAAGCCTACTACCCCGCCGGATCCGCCGCTGTCTCAGGAGAACAGCAGCATCAGCACGATGCGGCGGCTGCCGCGGCCATCGCTGAGGAACAAGGTAATCAACAATAGCTACTAGCATCCATCGATGTATCCAAACGTAGTCTTCTTTTGTCCGTGTGCACGTCGGATGGCGAGAGCATAGCACGCGCGCCTTCTGCTAGATGAATCGCAGCCATTTGGAAACTTCGAATCATCCAAACACGTCGATATAACTACTAATTTCCTTGTTTTCTTCATCCTAATATGTGGCGAGCTTAATTTAGTGGTAAAATGGCTGTATAAACTCTGGAGAGAAGTACAATAATTTTGCCTGTTTCGTGCACGAAGATACGCTAGCAGCGAAGTGCTTAAATTCAACTCCTCTCCTTATTCAATCCTGCACGAATATTTTATGCGCATACGTACGTGTATAAGTGTATTACGTTAGCTCTGACGAAACCACAAATTAATTATCAATTGTGTTCATCTGGGGGATGTGGCTGCAGGGAGGAAGCGGCACTACAGAGGGGTGAGGCAGCGGCCATGGGGAAAGTGGGCGGCGGAGATCCGGGACCCCAAGAAAGCGGCTCGTGTGTGGCTCGGCACCTTTGACACGGCTGAGGACGCCGCCATCGCCTACGACGAAGCGGCGCTGCGCTTCAAGGGCACCAAGGCCAAGCTCAACTTCCCCGAGCGCGTCCAGGGACGCACCGACCTCGGCTTCGTTGTCACGCGCGGCATACCCGACAGATTGCAGCAACAACAACACTACCCCGCCACCGTGGGGGCGCCGGCAATGCGGCCACCGCCGCACCAGCTGCAGACCGTGGTGCCGTACCCTGACCTCATGCGGTATGCACGGCTGTTGCAGGGCGCTGGCAGTGCCGGGGGCGCTGTCAACCTGCCGTTCGGCGCCATGTCGCCCCCGTCGATGTCCTCGTCGGCGCCGCACATACTCGACTTCTCGACACAGCAGCTCATCCGAGTGAGCCCGGCGTCTCCCGCGGCGGCAATGTCGAGCTCAGGCACAACGGGGCCGTCCACCTCATCGTCCACGACTACGGCATCGTCGCCAGGTGCTGCATGGCCGTACACTGGGGAGCACAAAAATAATAAAGACTCGTGAGAGATCAATGGATCGAAGGTGCTTGCGATCCATCGGGACATGTCTTAGCAGTAGATGAGGAATACGCATGCATTCATCATGCAaTAAGGTGGGTAAGTGTTGgTCAaTTAATTTTGCTGGTGAACATTTCTTTCCTTCTTCTCATCCCCAAATTTGTTTATCTAAAGCTCTTGTTTTGTTTATTTTTACTTTAATAATGGGTGATTTTTTTTGGCGGGTAATGGGTGACTCTTAACTAGCTAGGTACTAGTTGTACTAAATTAGTGACAAGTAACATAGAACAGAGGGAGACCTTGTTCCTTGGTCTAATCGTTTATCGTTTC

***Hap-2D-1*:**

CAGACTTCTCCTTCTATCACATCCCCTAGCTATCATTAAAGGCGTTCAGAAGAGCTCGTCTCCTCTCTCTCCCTCCCTCCGCGTTCTTATCAGTACGTTGTCCGCGCCTAGGCACCAAAGTCCAAAGCAACAGCCATAGCTCGATCTCGATCCCCGGCGCGACGAAAGAAAAAGAAGCGGCGGCAGGTCGACAGGTCGATCAACTAAGGTGGATCCCCGGAGGCATGGGAAGAGGCCCCTACCCGCCGACGAGGAGGAGGAACAGCCGCCACCGCCGCCGTCAGCACCCAAGCACGAGCAGGTGGAGGAGCAGCCGTATCACCACCTCATCGCGCACGCTCTGCAGCAGCAAGGAGCTGCCAGCGCCGGCGGAAGCCCGGGAGCAGATGTGGCCGACCCTTCCCCGTCACCGGAGGCGTACGCGCAGTACTACTACTCGGCGCGCGCCGACCACGACGCCACCGCCATGGTCTCCGCTCTGTCCCACGTCATCCGCGCCACACCGGACCAGCAACAAGCCTACTACCCCGCCGGATCCGCCGCTGTCTCAGGAGAACAGCAGCATCAGCACGATGCGGCGGCTGCAGCGGCCATCGCTGAGGAACAAGGTAATCAACAATACCTACTAGCATCCATCCATGTATCCAAACGTAGTCTTCTTTTGTCCGTGTGCACGTCGGATGTCGAGAGCATAGCACGCGCGCCTTCTGCTAGATGAATCGCAGCCATTTGGAAACGTCGAATCATCCAAACACGTCGATATAACTACTAATTTCCTTGTTTTCTTCATCCTAATATGTGGCGAGCTTAATTTAGTGGTAAAATGGCTGTATAAACTCTGGAGAGAAGTACAATAATTTTGCCTGTTTCGTGCACGAAGATACGCCAGCAGCGAAGTGCTTAAATTCAACTCCTCGCCTTATTCAATCCTGCACGAATATTTTATGCGCATACGTACGTGTATAAGTGTATTACGTTAGCTCTGACGAAACCACAAATTAATTATCAATTGTGTTCGGATGTGGCTGCAGGGAGGAAGCGGCACTACAGAGGGGTGAGGCAGCGGCCATGGGGAAAGTGGGCGGCGGAGATCCGGGACCCCAAGAAAGCGGCTCGTGTGTGGCTCGGCACCTTTGACACGGCTGAGGACGCCGCCATCGCCTACGACGAAGCGGCGCTGCGCTTCAAGGGCACCAAGGCCAAGCTCAACTTCCCCGAGCGCGTCCAGGGACGCACCGACCTCGGCTTCGTCGTCACGCGCGGCATACCCGACAGATTGCAGCAACAACAACAGTACCCCGCCACCGTGGGGGCGCCGGCAATGCGGCCACTGCCGCACCAGCAGCAGACCGTGGTGCCGTACCCTGACCTCATGCGGTATGCACAGCTGTTGCAGGGCGCTGGCAGTGCCGGGAGCGCCGTCAACCTGCCGTTCGGCGCCATGTCGCCCCCGTCGATGTCCTCGTCGTCGCCGCACATACTCGACTTCTCGACACAGCAGCTCATCCGAGTGAGCCCGACGTCTCCCGCGGCGGCTATATCGAGCTCAGGCACAACGGGGCCGTCCACCTCATCGTCCACGACTACGGCATCGTCGCCAGGTGCTGCATGGCCGTACACTGGGGAGCACAAAAATAATAAAGACTCGTGAGAGATCAGTGGATCGAAGGTGCTTGCGATCCATCGGGACATGTCTTAGCAGTAGATGAGGAATACGCATGCATTCATCATGCAATAAGGTGGGTAAGTGTTGGTCAATTGATTTTGCTGGTGAACATTTCTTTCCTTCTTCTGATCCCCAAATTTGTTCATCTAAATCTCTTGTTTTGTTTA

***Hap-2D-2*:**

CAGACTTCTCCTTCTATCACATCCCCTAGCTATCATTAAAGGCGTTCAGAAGAGCTCGTCTCCTCTCTCTCCCTCCCTCCGCGTTCTTATCAGTACGTTGTCCGCGCCTAGGCACCAAAGTCCAAAGCAACAGCCATAGCTCGATCTCGATCCCCGGCGCGACGAAAGAAAAAGAAGCGGCGGCAGGTCGACAGGTCGATCAACTAAGGTGGATCCCCGGAGGCATGGGAAGAGGCCCCTACCCGCCGACGAGGAGGAGGAACAGCCGCCACCGCCGCCGTCAGCACCCAAGCACGAGCAGGTGGAGGAGCAGCCGTATCACCACCTCATCGCGCACGCTCTGCAGCAGCAAGGAGCTGCCAGCGCCGGCGGAAGCCCGGGAGCAGATGTGGCCGACCCTTCCCCGTCACCGGAGGCGTACGCGCAGTACTACTACTCGGCGCGCGCCGACCACGACGCCACCGCCATGGTCTCCGCTCTGTCCCACGTCATCCGCGCCACACCGGACCAGCAACAAGCCTACTACCCCGCCGGATCCGCCGCTGTCTCAGGAGAACAGCAGCATCAGCACGATGCGGCGGCTGCAGCGGCCATCGCTGAGGAACAAGGTAATCAACAATACCTACTAGCATCCATCCATGTATCCAAACGTAGTCTTCTTTTGTCCGTGTGCACGTCGGATGTCGAGAGCATAGCACGCGCGCCTTCTGCTAGATGAATCGCAGCCATTTGGAAACGTCGAATCATCCAAACACGTCGATATAACTACTAATTTCCTTGTTTTCTTCATCCTAATATGTGGCGAGCTTAATTTAGTGGTAAAATGGCTGTATAAACTCTGGAGAGAAGTACAATAATTTTGCCTGTTTCGTGCACGAAGATACGCCAGCAGCGAAGTGCTTAAATTCAACTCCTCGCCTTATTCAATCCTGCACGAATATTTTATGCGCATACGTACGTGTATAAGTGTATTACGTTAGCTCTGACGAAACCACAAATTAATTATCAATTGTGTTCGGATGTGGCTGCAGGGAGGAAGCGGCACTACAGAGGGGTGAGGCAGCGGCCATGGGGAAAGTGGGCGGCGGAGATCCGGGACCCCAAGAAAGCGGCTCGTGTGTGGCTCGGCACCTTTGACACGGCTGAGGACGCCGCCATCGCCTACGACGAAGCGGCGCTGCGCTTCAAGGGCACCAAGGCCAAGCTCAACTTCCCCGAGCGCGTCCAGGGACGCACCGACCTCGGCTTCGTCGTCACGCGCGGCATACCCGACAGATTGCAGCAACAACAACAGTACCCCGCCACCGTGGGGGCGCCGGCAATGCGGCCACTGCCGCACCAGCAGCAGACCGTGGTGCCGTACCCTGACCTCATGCGGTATGCACAGCTGTTGCAGGGCGCTGGCAGTGCCGGGGGCGCCGTCAACCTGCCGTTCGGCGCCATGTCGCCCCCGTCGATGTCCTCGTCGTCGCCGCACATACTCGACTTCTCGACACAGCAGCTCATCCGAGTGAGCCCGACGTCTCCCGCGGCGGCTATATCGAGCTCAGGCACAACGGGGCCGTCCACCTCATCGTCCACGACTACGGCATCGTCGCCAGGTGCTGCATGGCCGTACACTGGGGAGCACAAAAATAATAAAGACTCGTGAGAGATCAGTGGATCGAAGGTGCTTGCGATCCATCGGGACATGTCTTAGCAGTAGATGAGGAATACGCATGCATTCATCATGCAATAAGGTGGGTAAGTGTTGGTCAATTGATTTTGCTGGTGAACATTTCTTTCCTTCTTCTGATCCCCAAATTTGTTCATCTAAATCTCTTGTTTTGTTTA

***Hap-2D-3*:**

CAGACTTCTCCTTCTATCACATCCCCTAGCTATCATTAAAGGCGTTCAGAAGAGCTCGTCTCCTCTCTCTCCCTCCCTCCGCGTTCTTATCAGTACGTTGTCCGCGCCTAGGCACCAAAGTCCAAAGCAACAGCCATAGCTCGATCTCGATCCCCGGCGCGACGAAAGAAAAAGAAGCGGCGGCAGGTCGACAGGTCGATCAACTAAGGTGGATCCCCGGAGGCATGGGAAGAGGCCCCTACCCGCCGACGAGGAGGAGGAACAGCCGCCACCGCCGCCGTCAGCACCCAAGCACGAGCAGGTGGAGGAGCAGCCGTATCACCACCTCATCGCGCACGCTCTGCAGCAGCAAGGAGCTGCCAGCGCCGGCGGAAGCCCGGGAGCAGATGTGGCCGACCCTTCCCCGTCACCGGAGGCGTACGCGCAGTACTACTACTCGGCGCGCGCCGACCACGACGCCACCGCCATGGTCTCCGCTCTGTCCCACGTCATCCGCGCCACACCGGACCAGCAACAAGCCTACTACCCCGCCGGATCCGCCGCTGTCTCAGGAGAACAGCAGCATCAGCACGATGCGGCGGCTGCAGCGGCCATCGCTGAGGAACAAGGTAATCAACAATACCTACTAGCATCCATCCATGTATCCAAACGTAGTCTTCTTTTGTCCGTGTGCACGTCGGATGTCGAGAGCATAGCACGCGCGCCTTCTGCTAGATGAATCGCAGCCATTTGGAAACGTCGAATCATCCAAACACGTCGATATAACTACTAATTTCCTTGTTTTCTTCATCCTAATATGTGGCGAGCTTAATTTAGTGGTAAAATGGCTGTATAAACTCTGGAGAGAAGTACAATAATTTTGCCTGTTTCGTGCACGAAGATACGCCAGCAGCGAAGTGCTTAAATTCAACTCCTCGCCTTATTCAATCCTGCACGAATATTTTATGCGCATACGTACGTGTATAAGTGTATTACGTTAGCTCTGACGAAACCACAAATTAATTATCAATTGTGTTCGGATGTGGCTGCAGGGAGGAAGCGGCACTACAGAGGGGTGAGGCAGCGGCCATGGGGAAAGTGGGCGGCGGAGATCCGGGACCCCAAGAAAGCGGCTCGTGTGTGGCTCGGCACCTTTGACACGGCTGAGGACGCCGCCATCGCCTACGACGAAGCGGCGCTGCGCTTCAAGGGCACCAAGGCCAAGCTCAACTTCCCCGAGCGCGTCCAGGGACGCACCGACCTCGGCTTCGTCGTCACGCGCGGCATACCCGACAGATTGCAGCAACAACAACAGTGCCCCGCCACCGTGGGGGCGCCGGCAATGCGGCCACTGCCGCACCAGCAGCAGACCGTGGTGCCGTACCCTGACCTCATGCGGTATGCACAGCTGTTGCAGGGCGCTGGCAGTGCCGGGGGCGCCGTCAACCTGCCGTTCGGCGCCATGTCGCCCCCGTCGATGTCCTCGTCGTCGCCGCACATACTCGACTTCTCGACACAGCAGCTCATCCGAGTGAGCCCGACGTCTCCCGCGGCGGCTATATCGAGCTCAGGCACAACGGGGCCGTCCACCTCATCGTCCACGACTACGGCATCGTCGCCAGGTGCTGCATGGCCGTACACTGGGGAGCACAAAAATAATAAAGACTCGTGAGAGATCAGTGGATCGAAGGTGCTTGCGATCCATCGGGACATGTCTTAGCAGTAGATGAGGAATACGCATGCATTCATCATGCAATAAGGTGGGTAAGTGTTGGTCAATTGATTTTGCTGGTGAACATTTCTTTCCTTCTTCTGATCCCCAAATTTGTTCATCTAAATCTCTTGTTTTGTTTA

**Additional file 3: Figure S2.** Nucleotide sequences of *TaERF8-2A*, *TaERF8-2B* and *TaERF8-2D* haplotypes*.*
