# Supplementary material for: Identification of a novel ERF gene, TaERF8, associated with plant height and yield in wheat
Source: BMC Plant Biol. 2020 Jun 8;20:263. doi: 10.1186/s12870-020-02473-6 (PMC7282131; doi:10.1186/s12870-020-02473-6)
Supplement: Supplementary file 4 — Additional file 4: Table S2. The information of sample set 1 and their genotypes of TaERF8-2B. [file 12870_2020_2473_MOESM4_ESM.docx]

**Additional file 4: Table S2.** The information of sample set 1 and their genotypes of *TaERF8-2B*

| Number | Accession | Type | Allele | Number | Accession | Type | Allele |
| --- | --- | --- | --- | --- | --- | --- | --- |
| 1 | Chadianhong | L | --- | 185 | Zhongluo08-2 | M | CTC |
| 2 | Dingxinzhai | L | --- | 186 | Zhongluo08-3 | M | CTC |
| 3 | Xiaobaimai | L | --- | 187 | Zhongmai13 | M | CTC |
| 4 | Heputou | L | --- | 188 | Zhongmai175 | M | CTC |
| 5 | Huajiechangbaican | L | --- | 189 | Zhongmai415 | M | CTC |
| 6 | Jiyumai | L | --- | 190 | Haocheng9411 | M | CTC |
| 7 | Youmangbaifu | L | --- | 191 | Heifu84S1378 | M | CTC |
| 8 | Baituzitou | L | --- | 192 | Huabei187 | M | CTC |
| 9 | Banjiemang | L | --- | 193 | Huaichuan916 | M | CTC |
| 10 | Dakoumai | L | --- | 194 | Huaimai22 | M | CTC |
| 11 | Fumai | L | --- | 195 | Jichun1016 | M | CTC |
| 12 | Hongquanmang | L | --- | 196 | Jimai19 | M | CTC |
| 13 | Huangguaxian | L | --- | 197 | Jimai20 | M | CTC |
| 14 | Louguding | L | --- | 198 | Jimai21 | M | CTC |
| 15 | Sanyuehuang | L | --- | 199 | Jimai22 | M | CTC |
| 16 | Xiaofoshou | L | --- | 200 | Jinan13 | M | CTC |
| 17 | Youzimai | L | --- | 201 | Jining12 | M | CTC |
| 18 | Youzitou | L | --- | 202 | Jining13 | M | CTC |
| 19 | Youmangshaogudan | L | --- | 203 | Jining16 | M | CTC |
| 20 | Lianglaiyoubaipixiaomai | L | --- | 204 | Ji5265 | M | CTC |
| 21 | Caijiangmai | L | --- | 205 | Jimai36 | M | CTC |
| 22 | Hongkejiang | L | --- | 206 | Jimai6 | M | CTC |
| 23 | Huanxiangguo | L | --- | 207 | Jiaomai266 | M | CTC |
| 24 | Yangmai | L | --- | 208 | Jinshajiang1 | M | CTC |
| 25 | Kashibaipi | L | --- | 209 | Jinmai2148 | M | CTC |
| 26 | Yutiandaomaizi | L | --- | 210 | Jing411 | M | CTC |
| 27 | Baicimai | L | CTC | 211 | Jingdong8 | M | CTC |
| 28 | Baihuamai | L | CTC | 212 | Jinghua2 | M | CTC |
| 29 | Baimaizi | L | CTC | 213 | Jingken49 | M | CTC |
| 30 | Baipu | L | CTC | 214 | Jingshuang16 | M | CTC |
| 31 | baixiaomai | L | CTC | 215 | Jingyang60 | M | CTC |
| 32 | Baiyoumai | L | CTC | 216 | Kaimai21 | M | CTC |
| 33 | Bendimai | L | CTC | 217 | Kanghuixianhong | M | CTC |
| 34 | Bendixiaomai | L | CTC | 218 | Kenong199 | M | CTC |
| 35 | Changgongfangxingmai | L | CTC | 219 | Kenhong14 | M | CTC |
| 36 | Changmangyingkemai | L | CTC | 220 | Laizhou953 | M | CTC |
| 37 | Chiyacao | L | CTC | 221 | Lankao90 | M | CTC |
| 38 | Dabaimai | L | CTC | 222 | liangxing66 | M | CTC |
| 39 | Dahongmai | L | CTC | 223 | Liangxing99 | M | CTC |
| 40 | Damoxu | L | CTC | 224 | Liaomai16 | M | CTC |
| 41 | Dazibai | L | CTC | 225 | Linkang5027 | M | CTC |
| 42 | Gezhoumai | L | CTC | 226 | Linmai2 | M | CTC |
| 43 | Heshangmai | L | CTC | 227 | Linmai3 | M | CTC |
| 44 | Heshangtou | L | CTC | 228 | Lumai14 | M | CTC |
| 45 | Hongheshangtou | L | CTC | 229 | Lumai1 | M | CTC |
| 46 | Honghuazao | L | CTC | 230 | Luyuan301 | M | CTC |
| 47 | Hongkeyoumang | L | CTC | 231 | Lunxuan987 | M | CTC |
| 48 | Hongmai | L | CTC | 232 | Luomai23 | M | CTC |
| 49 | Hongmangyouzimai | L | CTC | 233 | Luomai26 | M | CTC |
| 50 | Hongqiangchang | L | CTC | 234 | Mianmai1403 | M | CTC |
| 51 | Hongxiuzi | L | CTC | 235 | Mianmai185 | M | CTC |
| 52 | Huxumai | L | CTC | 236 | Mianmai37 | M | CTC |
| 53 | Huoqiu | L | CTC | 237 | Mianmai39 | M | CTC |
| 54 | Huoshaotian | L | CTC | 238 | Mianmai40 | M | CTC |
| 55 | Jiangdongmen | L | CTC | 239 | Mianmai41 | M | CTC |
| 56 | Jiangmai | L | CTC | 240 | Mianmai43 | M | CTC |
| 57 | Kangdingxiaomai | L | CTC | 241 | Mianmai45 | M | CTC |
| 58 | Kangmai | L | CTC | 242 | Mianmai46 | M | CTC |
| 59 | Lanhuamai | L | CTC | 243 | Mianmai48 | M | CTC |
| 60 | Lanxizaoxiaomai | L | CTC | 244 | Mianyang15 | M | CTC |
| 61 | Laomai | L | CTC | 245 | Mianyang19 | M | CTC |
| 62 | Liuzhutou | L | CTC | 246 | Mianyang20 | M | CTC |
| 63 | Makou | L | CTC | 247 | Mianyang38 | M | CTC |
| 64 | Mazhamai | L | CTC | 248 | Mianyang79-2 | M | CTC |
| 65 | Meiqianwu | L | CTC | 249 | Mianyang86-11 | M | CTC |
| 66 | Nuomai | L | CTC | 250 | Neijiang31 | M | CTC |
| 67 | Paozimai | L | CTC | 251 | Neixiang188 | M | CTC |
| 68 | Qigongmai | L | CTC | 252 | Nanda2419 | M | CTC |
| 69 | Ronganxiaomai | L | CTC | 253 | Nanda96co76 | M | CTC |
| 70 | Sankecun | L | CTC | 254 | Ningchun10 | M | CTC |
| 71 | Sanyuanmai | L | CTC | 255 | Ningchun13 | M | CTC |
| 72 | Shanhongmai | L | CTC | 256 | Xinchun8 | M | CTC |
| 73 | Shanmai | L | CTC | 257 | Xinkehan9 | M | CTC |
| 74 | Shanglingxiaomai | L | CTC | 258 | Xinmai26 | M | CTC |
| 75 | Sifangmai | L | CTC | 259 | Xinmai9 | M | CTC |
| 76 | Tumangmai | L | CTC | 260 | Xinxiang9178 | M | CTC |
| 77 | Tumai | L | CTC | 261 | Xinyang12 | M | CTC |
| 78 | Tuotuomai | L | CTC | 262 | Xingyi4 | M | CTC |
| 79 | Wumangmai | L | CTC | 263 | Xinmai1 | M | CTC |
| 80 | Wuyimai | L | CTC | 264 | Xuzhou14 | M | CTC |
| 81 | Wugongmai | L | CTC | 265 | Xuke718 | M | CTC |
| 82 | Xiaobaimang | L | CTC | 266 | Xunong7 | M | CTC |
| 83 | Xiaoziganzi | L | CTC | 267 | Yannong15 | M | CTC |
| 84 | Xinganxiaomai | L | CTC | 268 | Yannong22 | M | CTC |
| 85 | Yuqiumai | L | CTC | 269 | Yannong23 | M | CTC |
| 86 | Yunnanxiaomai3 | L | CTC | 270 | Yannong24 | M | CTC |
| 87 | Chinease spring | L | CTC | 271 | Yan893xuan | M | CTC |
| 88 | Zijihong | L | CTC | 272 | Yanzhan4110 | M | CTC |
| 89 | Zipi | L | CTC | 273 | Yangmai12 | M | CTC |
| 90 | Changfeng4 | M | --- | 274 | Yangmai13 | M | CTC |
| 91 | Changfeng6 | M | --- | 275 | Yangmai14 | M | CTC |
| 92 | Hanxuan10 | M | --- | 276 | Yangmai15 | M | CTC |
| 93 | Hanxuan3 | M | --- | 277 | Yangmai16 | M | CTC |
| 94 | Jinmai47 | M | --- | 278 | Yangmai17 | M | CTC |
| 95 | Jingdong22 | M | --- | 279 | Yangmai18 | M | CTC |
| 96 | Nongda311 | M | --- | 280 | Yangmai 5 | M | CTC |
| 97 | Shimai18 | M | --- | 281 | Kenda 4 | M | CTC |
| 98 | Anhui11 | M | --- | 282 | Ningchun4 | M | CTC |
| 99 | Xiaoyan22 | M | --- | 283 | Ningmai12 | M | CTC |
| 100 | Hezuo2 | M | --- | 284 | Ningmai14 | M | CTC |
| 101 | Songhuajiang1 | M | --- | 285 | Ningmai15 | M | CTC |
| 102 | Longfu91B-569 | M | --- | 286 | Ningmai9 | M | CTC |
| 103 | Fuzhuang30 | M | --- | 287 | Ningnuomai1 | M | CTC |
| 104 | Heibao4 | M | --- | 288 | Nongda1108 | M | CTC |
| 105 | Jinmai54 | M | --- | 289 | Nonda211 | M | CTC |
| 106 | Jimai26 | M | --- | 290 | Pinchun16 | M | CTC |
| 107 | Shi4185 | M | --- | 291 | Pindong34 | M | CTC |
| 108 | Yanda1817 | M | --- | 292 | Pinyang181 | M | CTC |
| 109 | Yanzhan1 | M | CTC | 293 | Qingfeng1 | M | CTC |
| 110 | CA9722 | M | CTC | 294 | Rikaze54 | M | CTC |
| 111 | FS056 | M | CTC | 295 | Shannong12 | M | CTC |
| 112 | FS059 | M | CTC | 296 | Shannong15 | M | CTC |
| 113 | FS198 | M | CTC | 297 | Shannong1870 | M | CTC |
| 114 | R59 | M | CTC | 298 | Shan160 | M | CTC |
| 115 | Aifeng3 | M | CTC | 299 | Shan253 | M | CTC |
| 116 | AK58 | M | CTC | 300 | Shan354 | M | CTC |
| 117 | Ailiduo | M | CTC | 301 | Shanhan8675 | M | CTC |
| 118 | Anyang1 | M | CTC | 302 | Shanmai150 | M | CTC |
| 119 | Bainong3217 | M | CTC | 303 | Shanmai159 | M | CTC |
| 120 | Beijing0045 | M | CTC | 304 | Shannong7859 | M | CTC |
| 121 | Beijing837 | M | CTC | 305 | Shanyou225 | M | CTC |
| 122 | Beijing8 | M | CTC | 306 | Shangluo81(2)4-19-23 | M | CTC |
| 123 | Bima1 | M | CTC | 307 | Shengxuan3 | M | CTC |
| 124 | Bima4 | M | CTC | 308 | Shiluan02-1 | M | CTC |
| 125 | Cayazheda29 | M | CTC | 309 | Shi5093 | M | CTC |
| 126 | Changnong339-5-1 | M | CTC | 310 | Shijiazhuang8 | M | CTC |
| 127 | Changwei18 | M | CTC | 311 | Shimai12 | M | CTC |
| 128 | Chuan84-7045 | M | CTC | 312 | Shimai15 | M | CTC |
| 129 | Chuanmai36 | M | CTC | 313 | Shimai19 | M | CTC |
| 130 | Chuanmai8 | M | CTC | 314 | Shiyou17 | M | CTC |
| 131 | Chuannong16 | M | CTC | 315 | Shiyou20 | M | CTC |
| 132 | Chuanyu19 | M | CTC | 316 | Shuangji4 | M | CTC |
| 133 | Chuanyu20 | M | CTC | 317 | Sumai3 | M | CTC |
| 134 | Chuanyu21 | M | CTC | 318 | Sumai6 | M | CTC |
| 135 | Dian662-525-2 | M | CTC | 319 | Taizhong23 | M | CTC |
| 136 | Dong1-23 | M | CTC | 320 | Taixue12 | M | CTC |
| 137 | Dong1-32 | M | CTC | 321 | Taimai1 | M | CTC |
| 138 | Dong2-23 | M | CTC | 322 | Tainong18 | M | CTC |
| 139 | Dong2-8 | M | CTC | 323 | Taishan21 | M | CTC |
| 140 | Dong3-6 | M | CTC | 324 | Taishan23 | M | CTC |
| 141 | Emai11 | M | CTC | 325 | Taishan9818 | M | CTC |
| 142 | Emai12 | M | CTC | 326 | TengS15 | M | CTC |
| 143 | Emai15 | M | CTC | 327 | Tianping3 | M | CTC |
| 144 | Emai16 | M | CTC | 328 | Wan7107 | M | CTC |
| 145 | Emai17 | M | CTC | 329 | Wanyuan-66 | M | CTC |
| 146 | Emai18 | M | CTC | 330 | Wanmai31 | M | CTC |
| 147 | Emai19 | M | CTC | 331 | Wanmai33 | M | CTC |
| 148 | Exi84-1031 | M | CTC | 332 | Weimai6 | M | CTC |
| 149 | Fan6 | M | CTC | 333 | Weimai8 | M | CTC |
| 150 | Fengchan3 | M | CTC | 334 | Weimai4 | M | CTC |
| 151 | Fengdecunmai1 | M | CTC | 335 | Wenmai4 | M | CTC |
| 152 | Fengkang8 | M | CTC | 336 | Wenmai6 | M | CTC |
| 153 | Ganmai8 | M | CTC | 337 | Wennong6 | M | CTC |
| 154 | Gao38 | M | CTC | 338 | Xian8 | M | CTC |
| 155 | Gaoyou503 | M | CTC | 339 | Xichang76-9 | M | CTC |
| 156 | Gaoyuan338 | M | CTC | 340 | Xinong6028 | M | CTC |
| 157 | Gaoyuan602 | M | CTC | 341 | Xinong979 | M | CTC |
| 158 | Gaoyou2018 | M | CTC | 342 | Xianmai10 | M | CTC |
| 159 | Guomai301 | M | CTC | 343 | Xian83 | M | CTC |
| 160 | Han6172 | M | CTC | 344 | Xiaoshan2134 | M | CTC |
| 161 | Han99-6143 | M | CTC | 345 | Xiaoyan54 | M | CTC |
| 162 | Yuamai21 | M | CTC | 346 | Yangguang 851 | M | CTC |
| 163 | Yumai2 | M | CTC | 347 | Youbao | M | CTC |
| 164 | Yumai49 | M | CTC | 348 | Yumai18 | M | CTC |
| 165 | Yumai70-36 | M | CTC | 349 | Zhongyu9398 | M | CTC |
| 166 | Yumai8 | M | CTC | 350 | Zhongyun | M | CTC |
| 167 | Yunong949 | M | CTC | 351 | Zhou18 | M | CTC |
| 168 | Yuandong3 | M | CTC | 352 | Zhoumai16 | M | CTC |
| 169 | Yuanxie62 | M | CTC | 353 | Zhoumai22 | M | CTC |
| 170 | Yuanzhu | M | CTC | 354 | Zhoumai23 | M | CTC |
| 171 | Zaosui30 | M | CTC | 355 | Zhouyuan9369 | M | CTC |
| 172 | Zhenmai168 | M | CTC | 356 | Zimai12 | M | CTC |
| 173 | Zhenmai3 | M | CTC | 357 | Yanzhan1 | M | CTC |
| 174 | Zhenmai4 | M | CTC | 358 | Zhongren2 | M | CTC |
| 175 | Zhenmai5 | M | CTC | 359 | Zhongxin78 | M | CTC |
| 176 | Zhenmai6 | M | CTC | 360 | Zhongyou206 | M | CTC |
| 177 | Zhenmai9 | M | CTC | 361 | Zhongyou9507 | M | CTC |
| 178 | Zhengmai379 | M | CTC | 362 | Zhongyu12 | M | CTC |
| 179 | Zhengmai9023 | M | CTC | 363 | Kexin 9 | M | CTC |
| 180 | Zhengmai9201 | M | CTC | 364 | Keyi 26 | M | CTC |
| 181 | Zhongjiao1 | M | CTC | 365 | Kefeng 3 | M | CTC |
| 182 | Zhongjiao2 | M | CTC | 366 | Xinchun11 | M | CTC |
| 183 | Zhongjiao3 | M | CTC | 367 | Xinchun6 | M | CTC |
| 184 | Zhongluo08-1 | M | CTC |  |  |  |  |

L: Landraces; M: Modern varieties
