# Supplementary material for: Identification of a novel ERF gene, TaERF8, associated with plant height and yield in wheat
Source: BMC Plant Biol. 2020 Jun 8;20:263. doi: 10.1186/s12870-020-02473-6 (PMC7282131; doi:10.1186/s12870-020-02473-6)
Supplement: Supplementary file 5 — Additional file 5: Table S3. Association analysis of TaERF8-2B haplotypes and agronomic traits in nine environments. [file 12870_2020_2473_MOESM5_ESM.docx]

**Additional file 5: Table S3.** Association analysis of *TaERF8-2B* haplotypes with agronomic traits in nine environments

| Environment | HD (*P*-value) | TKW (*P*-value) | PH (*P*-value) |
| --- | --- | --- | --- |
| E1 | 3.0E-05** | 2.2E-07** | 9.68E-08** |
| E2 | 1.66E-05** | 1.14E-10** | 7.34E-11** |
| E3 | 4.45E-05** | 3.05E-07** | 6.21E-07** |
| E4 | 2.07E-05** | 6.55E-10** | 3.39E-08** |
| E5 | 6.93E-04** | 7.02E-14** | 2.44E-09** |
| E6 | 7.47E-06** | / | 4.11E-11** |
| E7 | 0.02* | 0.03* | 6.27E-10** |
| E8 | 5.77E-03** | 3.03E-07** | 3.75E-11** |
| E9 | 1.27E-06** | 3.58E-13** | 6.74E-11** |

HD, heading date; TKW, thousand kernel weight; PH, plant height; E1 to E9, the environments of 2012-BJ, 2012-XX, 2012-JZ, 2012-LY, 2014-XX, 2014-BJ, 2015-XX, 2015-BJ and 2015-JZ, respectively.*P < 0.05 and **P < 0.01, respectively.
