# Supplementary material for: Identification of a novel ERF gene, TaERF8, associated with plant height and yield in wheat
Source: BMC Plant Biol. 2020 Jun 8;20:263. doi: 10.1186/s12870-020-02473-6 (PMC7282131; doi:10.1186/s12870-020-02473-6)
Supplement: Supplementary file 6 — Additional file 6: Table S4. Summary of agronomical traits of sample set 1 in nine environments. [file 12870_2020_2473_MOESM6_ESM.docx]

**Additional file 6: Table S4.** Summary of agronomical traits of sample set 1 in nine environments

| Environment | Haplotypes | PH (cm) | HD (days) | TKW (g) |
| --- | --- | --- | --- | --- |
| E1 | *Hap-2B-1* | 109.07±3.29 | 30.35±0.63 | 32.36±1.31 |
|  | *Hap-2B-2* | 89.40±1.35 | 27.85±0.21 | 39.93±0.52 |
| E2 | *Hap-2B-1* | 114.83±3.63 | 25.50±0.61 | 33.73±1.03 |
|  | *Hap-2B-2* | 89.12±1.37 | 22.53±0.18 | 41.42±0.38 |
| E3 | *Hap-2B-1* | 116.09±4.08 | 27.51±0.70 | 35.82±1.03 |
|  | *Hap-2B-2* | 94.06±1.52 | 25.26±0.18 | 41.64±0.39 |
| E4 | *Hap-2B-1* | 121.65±3.78 | 22.17±0.57 | 32.06±1.17 |
|  | *Hap-2B-2* | 97.91±2.12 | 20.16±0.24 | 38.40±0.63 |
| E5 | *Hap-2B-1* | 109.58±2.32 | 16.61±0.75 | 38.66±0.97 |
|  | *Hap-2B-2* | 93.13±1.24 | 14.03±0.33 | 46.68±0.46 |
| E6 | *Hap-2B-1* | 105.44±2.65 | 32.32±0.78 | ／ |
|  | *Hap-2B-2* | 85.37±1.29 | 29.42±0.25 | ／ |
| E7 | *Hap-2B-1* | 113.96±2.47 | 20.95±0.54 | 30.81±1.97 |
|  | *Hap-2B-2* | 97.18±1.14 | 19.54±0.26 | 37.16±1.11 |
| E8 | *Hap-2B-1* | 106.08±3.22 | 37.86±0.55 | 32.53±0.99 |
|  | *Hap-2B-2* | 82.75±1.44 | 36.16±0.27 | 37.60±0.39 |
| E9 | *Hap-2B-1* | 111.71±2.24 | 22.16±0.57 | 34.30±0.72 |
|  | *Hap-2B-2* | 94.30±1.18 | 19.40±0.24 | 40.53±0.38 |

PH, plant height; HD, heading date; TKW, thousand kernel weight; E1 to E9, the environments of 2012-BJ, 2012-XX, 2012-JZ, 2012-LY, 2014-XX, 2014-BJ, 2015-XX, 2015-BJ and 2015-JZ, respectively. Heading Date (days from April 1^st^). Data represents Mean ± *SE.*
