# Supplementary material for: Identification of a novel ERF gene, TaERF8, associated with plant height and yield in wheat
Source: BMC Plant Biol. 2020 Jun 8;20:263. doi: 10.1186/s12870-020-02473-6 (PMC7282131; doi:10.1186/s12870-020-02473-6)
Supplement: Supplementary file 7 — Additional file 7: Table S5. The information of sample set 2 and their genotypes of TaERF8-2B. [file 12870_2020_2473_MOESM7_ESM.docx]

**Additional file 7: Table S5.** The information of sample set 2 and their genotypes of *TaERF8-2B*

| Number | Accession | Type | Allele | Origin | Number | Accession | Type | Allele | Origin |
| --- | --- | --- | --- | --- | --- | --- | --- | --- | --- |
| 1 | PI 266841 | WEW | --- | United Kingdom | 71 | PI 487260 | WEW | CTC | Syria |
| 2 | PI 289602 | WEW | --- | United Kingdom | 72 | PI 487261 | WEW | CTC | Syria |
| 3 | PI 330543 | WEW | --- | United Kingdom | 73 | PI 487253 | WEW | CTC | Syria |
| 4 | PI 343446 | WEW | --- | Israel | 74 | PI 487254 | WEW | CTC | Syria |
| 5 | PI 346783 | WEW | --- | Hungary | 75 | PI 487255 | WEW | CTC | Syria |
| 6 | PI 352324 | WEW | --- | Lebanon | 76 | PI 487256 | WEW | CTC | Syria |
| 7 | PI 352327 | WEW | --- | Switzerland | 77 | PI 487257 | WEW | CTC | Syria |
| 8 | PI 355457 | WEW | --- | Germany | 78 | PI 487258 | WEW | CTC | Syria |
| 9 | PI 355458 | WEW | --- | Germany | 79 | PI 487259 | WEW | CTC | Syria |
| 10 | PI 414721 | WEW | --- | Israel | 80 | PI 487262 | WEW | CTC | Syria |
| 11 | PI 466949 | WEW | --- | Israel | 81 | PI 487263 | WEW | CTC | Syria |
| 12 | PI 466950 | WEW | --- | Israel | 82 | PI 487264 | WEW | CTC | Syria |
| 13 | PI 466951 | WEW | --- | Israel | 83 | PI 428018 | WEW | CTC | Turkey |
| 14 | PI 466953 | WEW | --- | Israel | 84 | PI 428019 | WEW | CTC | Turkey |
| 15 | PI 355459 | WEW | CTC | Armenia | 85 | PI 428020 | WEW | CTC | Turkey |
| 16 | PI 352323 | WEW | CTC | Asia Minor | 86 | PI 428021 | WEW | CTC | Turkey |
| 17 | PI 272582 | WEW | CTC | Hungary | 87 | PI 428023 | WEW | CTC | Turkey |
| 18 | PI 428016 | WEW | CTC | Iran | 88 | PI 428025 | WEW | CTC | Turkey |
| 19 | PI 300989 | WEW | CTC | Israel | 89 | PI 428024① | WEW | CTC | Turkey |
| 20 | PI 316905 | WEW | CTC | Israel | 90 | PI 554581 | WEW | CTC | Turkey |
| 21 | PI 415147 | WEW | CTC | Israel | 91 | PI 470736 | WEW | CTC | Turkey |
| 22 | PI 415148 | WEW | CTC | Israel | 92 | PI 554584 | WEW | CTC | Turkey |
| 23 | PI 415149 | WEW | CTC | Israel | 93 | PI 428017 | WEW | CTC | Turkey |
| 24 | PI 415151 | WEW | CTC | Israel | 94 | PI 428145 | WEW | CTC | Turkey |
| 25 | PI 414720 | WEW | CTC | Israel | 95 | PI 560872 | WEW | CTC | Turkey |
| 26 | PI 466979 | WEW | CTC | Israel | 96 | PI 560873 | WEW | CTC | Turkey |
| 27 | PI 466980-W | WEW | CTC | Israel | 97 | PI 560874 | WEW | CTC | Turkey |
| 28 | PI 466980-B | WEW | CTC | Israel | 98 | PI 560875 | WEW | CTC | Turkey |
| 29 | PI 466981 | WEW | CTC | Israel | 99 | PI 560876 | WEW | CTC | Turkey |
| 30 | PI 466982 | WEW | CTC | Israel | 100 | PI 560877 | WEW | CTC | Turkey |
| 31 | PI 466983 | WEW | CTC | Israel | 101 | PI 428085 | WEW | CTC | Turkey |
| 32 | PI 466984 | WEW | CTC | Israel | 102 | PI 554580 | WEW | CTC | Turkey |
| 33 | PI 466985 | WEW | CTC | Israel | 103 | PI 554582 | WEW | CTC | Turkey |
| 34 | PI 466986 | WEW | CTC | Israel | 104 | PI 554583 | WEW | CTC | Turkey |
| 35 | PI 466987 | WEW | CTC | Israel | 105 | PI 428086 | WEW | CTC | Turkey |
| 36 | PI 466988 | WEW | CTC | Israel | 106 | PI 190919 | WEW | --- | Spain |
| 37 | PI 300990 | WEW | CTC | Israel | 107 | PI 233288 | WEW | --- | Israel |
| 38 | PI 414718 | WEW | CTC | Israel | 108 | PI 94673 | DEW | CTC | Armenia |
| 39 | PI 414719 | WEW | CTC | Israel | 109 | PI 94618 | DEW | CTC | Belarus |
| 40 | PI 414722 | WEW | CTC | Israel | 110 | PI 94649 | DEW | CTC | Czechoslovakia |
| 41 | PI 428015 | WEW | CTC | Israel | 111 | PI 94630 | DEW | CTC | Ethiopia |
| 42 | PI 471035 | WEW | CTC | Israel | 112 | PI 94632 | DEW | CTC | Ethiopia |
| 43 | PI 471036 | WEW | CTC | Israel | 113 | PI 195723 | DEW | CTC | Ethiopia |
| 44 | PI 471037 | WEW | CTC | Israel | 114 | PI 194375 | DEW | CTC | Ethiopia |
| 45 | PI 471038 | WEW | CTC | Israel | 115 | PI 196099 | DEW | CTC | Ethiopia |
| 46 | PI 428126 | WEW | CTC | Lebanon | 116 | PI 196100 | DEW | CTC | Ethiopia |
| 47 | PI 428127 | WEW | CTC | Lebanon | 117 | PI 191386 | DEW | CTC | Ethiopia |
| 48 | PI 428128 | WEW | CTC | Lebanon | 118 | PI 197259 | DEW | CTC | Ethiopia |
| 49 | PI 428129 | WEW | CTC | Lebanon | 119 | PI 197486 | DEW | CTC | Ethiopia |
| 50 | PI 428130 | WEW | CTC | Lebanon | 120 | PI 197494 | DEW | CTC | Ethiopia |
| 51 | PI 428131 | WEW | CTC | Lebanon | 121 | PI 193642 | DEW | CTC | Ethiopia |
| 52 | PI 428125 | WEW | CTC | Lebanon | 122 | PI 193873 | DEW | CTC | Ethiopia |
| 53 | PI 352322 | WEW | CTC | Lebanon | 123 | PI 193883 | DEW | CTC | Ethiopia |
| 54 | PI 470978 | WEW | CTC | Lebanon | 124 | PI 194041 | DEW | CTC | Ethiopia |
| 55 | PI 470979 | WEW | CTC | Lebanon | 125 | PI 194042 | DEW | CTC | Ethiopia |
| 56 | PI 470980 | WEW | CTC | Lebanon | 126 | PI 196905 | DEW | CTC | Ethiopia |
| 57 | PI 470981 | WEW | CTC | Lebanon | 127 | Cltr 14838 | DEW | CTC | Ethiopia |
| 58 | PI 470982 | WEW | CTC | Lebanon | 128 | PI 113961 | DEW | CTC | Georgia |
| 59 | PI 256029 | WEW | CTC | Spain | 129 | PI 101971 | DEW | CTC | India |
| 60 | PI 466927 | WEW | CTC | Syria | 130 | PI 94633 | DEW | CTC | Morocco |
| 61 | PI 466928 | WEW | CTC | Syria | 131 | PI 94669 | DEW | CTC | Russian |
| 62 | PI 466929 | WEW | CTC | Syria | 132 | PI 94660 | DEW | CTC | Russian |
| 63 | PI 466930 | WEW | CTC | Syria | 133 | PI 190922 | DEW | --- | Spain |
| 64 | PI 466931 | WEW | CTC | Syria | 134 | PI 154582 | DEW | --- | Taiwan |
| 65 | PI 466932 | WEW | CTC | Syria | 135 | PI 94738 | DEW | CTC | Ukraine |
| 66 | PI 470944 | WEW | CTC | Syria | 136 | PI 94656 | DEW | CTC | Yugoslavia |
| 67 | PI 470945 | WEW | CTC | Syria | 137 | PI 94657 | DEW | CTC | Yugoslavia |
| 68 | PI 470946 | WEW | CTC | Syria | 138 | PI 94671 | DEW | CTC | Afghanistan |
| 69 | PI 487251 | WEW | CTC | Syria | 139 | PI 94621 | DEW | CTC | Armenia |
| 70 | PI 487252 | WEW | CTC | Syria |  |  |  |  |  |

WEW: wild emmer wheat; DEW: domesticated emmer wheat.
