# Supplementary material for: Identification of a novel ERF gene, TaERF8, associated with plant height and yield in wheat
Source: BMC Plant Biol. 2020 Jun 8;20:263. doi: 10.1186/s12870-020-02473-6 (PMC7282131; doi:10.1186/s12870-020-02473-6)
Supplement: Supplementary file 8 — Additional file 8: Table S6. The frequencies of TaERF8-2B allelic variation in sample set 3. [file 12870_2020_2473_MOESM8_ESM.docx]

**Additional file 8: Table S6.** The frequencies (%) of *TaERF8-2B* allelic variation in sample set 3

| Zone | *Hap-2B-1* | *Hap-2B-2* | Total number |
| --- | --- | --- | --- |
| I | 4 (36.4) | 7 (63.6) | 11 |
| II | 12 (24) | 38 (76) | 50 |
| III | 17 (10.4) | 146(89.6) | 163 |
| IV | 5 (8) | 57 (92) | 62 |
| V | 4 (7) | 53 (93) | 57 |
| **Total** | **42(12.2)** | **301 (87.8)** | **343** |

I, Northeastern Spring Wheat Zone; II, Northern Winter Wheat Zone; III, Huanghuai River Winter Wheat Zone; IV, the middle and lower reaches of Yangtze River Winter Wheat Zone; V, Southwestern Winter Wheat Zone.
