# Supplementary material for: Identification of a novel ERF gene, TaERF8, associated with plant height and yield in wheat
Source: BMC Plant Biol. 2020 Jun 8;20:263. doi: 10.1186/s12870-020-02473-6 (PMC7282131; doi:10.1186/s12870-020-02473-6)
Supplement: Supplementary file 9 — Additional file 9: Figure S3. The expression levels of TaERF8-2B in overexpression transgenic wheat lines and wild-type plants. [file 12870_2020_2473_MOESM9_ESM.docx]

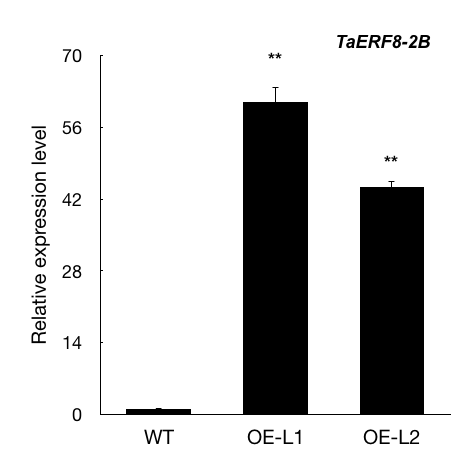


**Additional file 9: Figure S3.** The expression levels of *TaERF8-2B* in overexpression transgenic wheat lines and wild-type plants. WT: wild type; OE: *TaERF8-2B* overexpression transgenic wheat; data were means ± SD, ^**^*P* < 0.01 (Student’s *t* test); the experiment was performed independently three times with similar results.

.
